# Next-Generation Nanocarrier Platforms for RNA Vaccines: Advances in Formulation, Stability Engineering, and Translational Manufacturing Challenges

**DOI:** 10.3390/pharmaceutics18080909

**Published:** 2026-07-23

**Authors:** Mohannad M. Fallatah, Samiyah Al-Khaldi, Dimah K. Alrabiah, Ibrahim A. Alradwan, Mohammad N. Alkhrayef, Alhassan H. Aodah, Essam J. Alyamani, Esraa A. Aldkheil, Seham S. Alharthy, Najlaa A. Abualsaud, Yahya F. Jamous, Ahmad M. Aldossary

**Affiliations:** 1 Advanced Diagnostics and Therapeutics Institute, Health Sector, King Abdulaziz City for Science and Technology (KACST), Riyadh 12354, Saudi Arabia; mfallatah@kacst.gov.sa (M.M.F.); ialradwan@kacst.gov.sa (I.A.A.);; 2 Applied Genomics Technologies Institute, Health Sector, King Abdulaziz City for Science and Technology (KACST), Riyadh 11442, Saudi Arabia; salkhaldi@kacst.gov.sa; 3 Wellness and Preventive Medicine Institute, Health Sector, King Abdulaziz City for Science and Technology (KACST), Riyadh 11442, Saudi Arabia; dalrabiah@kacst.gov.sa (D.K.A.);; 4 Disability Research Institute, Health Sector, King Abdulaziz City for Science and Technology (KACST), Riyadh 11442, Saudi Arabia; mkhuryef@kacst.gov.sa; 5 Bioengineering Institute, Health Sector, King Abdulaziz City for Science and Technology (KACST), Riyadh 12354, Saudi Arabia; ealdkheil@kacst.gov.sa; 6 Department of Biology, College of Science, Princess Nourah bint Abdulrahman University, Riyadh 11671, Saudi Arabia; 446200541@pnu.edu.sa

**Keywords:** RNA vaccines, nanocarriers, RNA delivery, immune response, GMP

## Abstract

RNA vaccines have emerged as an attractive platform for treating infectious diseases, cancer immunotherapy, and personalized medicine; however, their clinical success depends on multiple factors, including efficient, stable, and scalable delivery systems. Because RNA molecules are highly sensitive to factors such as enzymatic degradation, oxidation, poor cellular uptake, and limited endosomal escape, nanocarrier platforms play essential roles in protecting RNA cargo and enabling effective intracellular delivery. The biological performance of RNA nanocarriers depends on efficient cellular uptake, endosomal escape, intracellular RNA delivery, biodistribution, and immune modulation. Comparative assessment emphasizes that lipid nanoparticles remain the most clinically mature approach, while nanostructured lipid carriers, polymeric systems, and exosome-based nanocarriers provide multiple benefits for stability, targeted delivery, biocompatibility, and/or controlled release. Translational challenges involving GMP manufacturing, batch reproducibility, regulatory expectations, and scale-up are considered critical for effective nano-based RNA vaccine delivery and are elaborated in this review. Emerging advances such as pKa-tuned ionizable lipids, ligand-targeted systems, stimuli-responsive nanocarriers, circular and self-amplifying RNA platforms, artificial intelligence-guided formulation design, and needle-free delivery technologies may further expand the safety, accessibility, and therapeutic potential of RNA vaccines. In this review, we highlight next-generation nanocarrier systems for RNA vaccines, with an emphasis on novel nanocarrier RNA vaccine delivery systems. Additionally, we evaluate stability engineering approaches that currently limit global vaccine distribution and the future of the nanocarrier platforms for RNA vaccines.

## 1. Introduction

The development of RNA vaccines has evolved significantly over the past six decades, representing one of the most transformative advancements in modern immunology. Messenger RNA (mRNA) was first discovered in 1961, and by 1969, researchers successfully produced proteins from isolated mRNA. Liposomes, which were developed in 1965, were later adapted for drug and vaccine delivery in the 1970s. In 1990, Wolff et al. demonstrated that direct intramuscular injection of in vitro-transcribed mRNA into mouse skeletal muscle resulted in detectable gene expression in vivo, providing the first evidence of successful mRNA delivery without viral vectors [1]. Building on these innovations, refined lipid nanoparticle (LNP) systems emerged in 2001, enabling the initiation of mRNA vaccine trials by 2013 [2]. In 2018, patisiran (Onpattro) became the first lipid nanoparticle (LNP)-based small interfering RNA (siRNA) therapeutic to receive regulatory approval, marking a significant milestone in RNA-based drug delivery [3]. The true breakthrough occurred in 2020, when mRNA vaccines for COVID-19 received emergency Food and Drug Administration (FDA) authorization, demonstrating high effectiveness and marking a pivotal milestone in vaccine technology [2,4].

mRNA vaccines work by encoding antigens that elicit both humoral and cell-mediated immune responses; however, the mRNA molecule itself is inherently unstable due to susceptibility to RNase degradation [2]. To enhance stability and translation efficiency, mRNA constructs incorporate 5′ capping, poly(A) tails, and optimized untranslated regions (UTRs), while LNP encapsulation protects the mRNA and enhances delivery into antigen-presenting cells (APCs) [2,4]. Further design elements such as the Kozak sequence, efficient promoters, and codon optimization contribute to improved antigen expression and vaccine efficiency [2]. Compared to traditional vaccines, mRNA vaccines offer numerous advantages, including rapid and cost-effective manufacturing, adaptability to emerging variants, and the ability to induce both antibody and CD8^+^ T cell responses, which are crucial for combating infectious diseases and hold significant potential in cancer immunotherapy [2,4]. Unlike DNA vaccines, mRNA functions directly in the cytoplasm, bypassing nuclear transcription and allowing faster antigen production with a reduced risk of genomic integration [4]. Additionally, LNPs serve not only as delivery agents but also as immune stimulators, enhancing humoral and cellular responses. Furthermore, double-stranded RNA (dsRNA) structures activate innate immune receptors such as TLR3 and MDA5, stimulating the MDA5–IFN-β signaling pathway and promoting cellular immunity [4]. Chemically modified nucleosides were shown to prevent Toll-like receptor activation by in vitro-transcribed RNA, whereas unmodified RNA stimulated TLR3, TLR7, and TLR8. However, the necessity and extent of nucleoside modification may vary depending on the intended therapeutic application, including mRNA-based CAR-T cell engineering [5]. The success of vaccines such as mRNA-1273 and BNT162b2, which achieved over 90% efficacy, validated RNA technology as a versatile platform for infectious disease prevention and cancer immunotherapy [4]. Their self-adjuvating properties and transient cytoplasmic activity further contribute to enhanced safety and strong immunogenicity, supporting their role in the advancement of next-generation vaccines [4].

Beyond SARS-CoV-2, recent phase 3 clinical trials have further validated the mRNA platform, demonstrating superior protection of a seasonal influenza mRNA vaccine compared with licensed standard-dose vaccines, as well as significant efficacy of an mRNA-based RSV vaccine in adults [6].

After the success of non-replicating mRNA during the COVID-19 pandemic, several next-generation RNA vaccine strategies have been developed, including self-amplifying RNA, trans-amplifying RNA, and circular RNA [7]. The mechanism of action of conventional mRNA release is direct encoding of the antigen, which is translated in the cytoplasm without intracellular replication, while self-amplifying RNA contains replicase genes that allow intracellular RNA amplification and reduce the vaccination dose drastically. However, the size of self-amplifying RNA constructs is large, which may affect the manufacturing and the quality of the induction of sufficient immune responses [8]. Trans-amplifying RNA, on the other hand, is designed to overcome these challenges; it has a split-vector system that divides replicase and antigen-encoding components into two distinct RNA fragments, reducing the size of the antigen-encoding construct, although efficient co-delivery of both RNA components is required [9]. Circular RNA is resistant to exonuclease-mediated degradation because it lacks free 5′ and 3′ ends, resulting in a longer period of protein expression, but its large-scale manufacturing remains under investigation [10]. These differences between RNA vaccine platforms directly influence nanocarrier composition and properties.

The rise of RNA-based therapeutics and vaccines has revealed the need for more advanced delivery systems capable of addressing persistent challenges such as molecular instability, degradation, and inefficient intracellular uptake. RNA interference (RNAi)-based strategies for targeting circular RNAs (circRNAs) also face off-target effects and difficulties in achieving precise tissue-specific delivery, underscoring the importance of developing new nanocarrier systems [11]. Among current platforms, LNPs represent the most developed delivery technology for nucleic acid-based vaccines and therapeutics. Typically composed of ionizable or cationic lipids, helper lipids, cholesterol, and PEG-modified lipids, LNPs enhance RNA stability, protect against enzymatic degradation, and improve cellular internalization [4,12]. Nonetheless, conventional LNPs and similar carriers face limitations related to stability, scalability, and targeted delivery efficiency, prompting ongoing innovation in nanocarrier design [13]. Nanotechnology plays a central role in overcoming these barriers by enhancing nucleic acid stability and facilitating effective transfection into APCs [14]. The development of next-generation nanocarriers aims to refine the physical and chemical characteristics of delivery systems to achieve greater precision, safety, and scalability while ensuring suitability for large-scale manufacturing and clinical implementation. Importantly, synthetic self-replicating RNAs (srRNAs) demonstrate how novel delivery formulations, such as LNPs or polymer-based systems, can replace traditional viral particles to improve safety and control [14]. Collectively, the rationale behind next-generation nanocarriers lies in enhancing the precision, efficacy, and manufacturability of RNA delivery systems to unlock their full immunological and therapeutic potential.

Despite major achievements in RNA vaccine development, various scientific and logistical challenges continue to impede their widespread use and long-term impact. While the mRNA-1273 (Moderna) and BNT162b2 (Pfizer) vaccines showed high protection against COVID-19, they were less effective in curbing community transmission, as neutralizing antibody titers declined rapidly and side effects were observed [15]. Moreover, these vaccines have struggled to induce strong mucosal immunity and functional secretory IgA (sIgA), both of which are essential for preventing upper respiratory infections [2]. Post-vaccination cases further revealed insufficient durability of immune protection, emphasizing the need to address challenges related to antigen mutations, safety, and cold-chain storage requirements [4]. Cancer-oriented mRNA vaccines also remain in early-stage clinical evaluation, showing limited therapeutic success so far [4]. To achieve improved efficacy and safety, further refinement of mRNA sequence design and delivery systems is essential [4]. However, the srRNA platforms introduce additional complications, including dose-limiting toxicities caused by impurities and LNP composition, suboptimal antibody titers, and occasional inflammatory responses at low doses [14]. Moreover, the inherent self-adjuvanticity of RNA can activate intracellular RNA sensors, leading to degradation or suppression of expression [12]. Optimization strategies such as 5′ capping, 3′ poly(A) tail modification, the inclusion of untranslated regions, and nucleoside substitutions can help mitigate these limitations [14]. Despite being nonintegrative, noninfectious, and well-tolerated, mRNA vaccines remain sensitive to storage conditions, requiring stringent cold-chain logistics that increase manufacturing and distribution costs [16]. In parallel, emerging circRNA vaccines present their own set of developmental challenges, as efforts continue to refine their design, synthesis, purification, and targeted delivery [16]. Overcoming these technical barriers will be essential for fully realizing the potential of mRNA, srRNA, and circRNA vaccines as next-generation immunotherapeutic platforms.

The aim of this review is to provide an in-depth analysis of RNA vaccine nanocarrier technologies, focusing on their design principles, formulation strategies, stability optimization, biological performance, and translational challenges. The review seeks to integrate current knowledge on lipid- and polymer-based systems while highlighting recent innovations that enhance RNA delivery efficiency, safety, and scalability. Structurally, the paper begins with an introduction outlining the background, rationale, and key challenges in RNA vaccine development, followed by a comprehensive overview of nanocarrier systems and formulation approaches. Subsequent sections address stability engineering, biological mechanisms, comparative analyses of carrier types, and considerations for manufacturing and regulatory translation. The review concludes with future directions and emerging technologies, providing a roadmap for advancing next-generation RNA vaccine platforms.

## 2. Structural Barriers and Diversity of RNA Delivery Nanocarrier Platforms

The clinical translation of RNA-based therapeutics and vaccines is highly dependent on advances in efficient delivery systems [17]. Advanced nanocarriers have greatly impacted the field of RNA therapeutics by overcoming structural instability, facilitating cellular membrane penetration, and mitigating immunogenicity challenges, which are commonly associated with naked RNA [18]. Moreover, RNA molecules are extremely vulnerable to degradation by extracellular ribonucleases, owing to their substantial size, and they possess a significant negative charge, which inhibits passive diffusion across cellular lipid membranes [19]. Similarly, administering unprotected RNA throughout the body leads to rapid elimination of the molecule and nonspecific distribution [20]. In addition to their physicochemical instability, unprotected RNA can trigger the innate immune response pathways, including some Toll-like receptors, such as TLR3, TLR7, and TLR8, as well as the cytosolic recognition receptors, including RIG-I and MDA5 [21,22]. Subsequently, this premature immune recognition ultimately leads to inflammation and decreased protein translation [23]. These challenges highlight the need for designing a delivery system that is capable of protecting RNAs from degradation, as well as releasing the cytosolic RNA effectively [24]. Therefore, the nanocarrier delivery system aims at overcoming the above-stated limitations by developing a stable structure based on nanoscale for protecting and transporting the RNA, along with its effective endosomal escape, and enabling targeted delivery specifically [20].

Typically, nanocarrier-mediated RNA transport involves several distinct physiological steps: structural protection against extracellular nucleases, distribution to target tissue either via systemic circulation or localized to the injection site for intramuscular and mucosal vaccines, cellular uptake via endocytosis, and ultimately endosomal escape into the cytosol [25]. In addition, the demand for systemic distribution of the nanocarrier is highly dependent on the administration route. In systemic drug delivery systems, the nanocarrier should remain in the blood circulation for an adequate period to be able to deliver its payload to target sites. On the other hand, RNA nanocarriers, which are used for local vaccination purposes, are normally intended to remain local to deliver the molecules to resident immune cells rather than to circulate systemically for a prolonged period [26].

**Figure 1:**
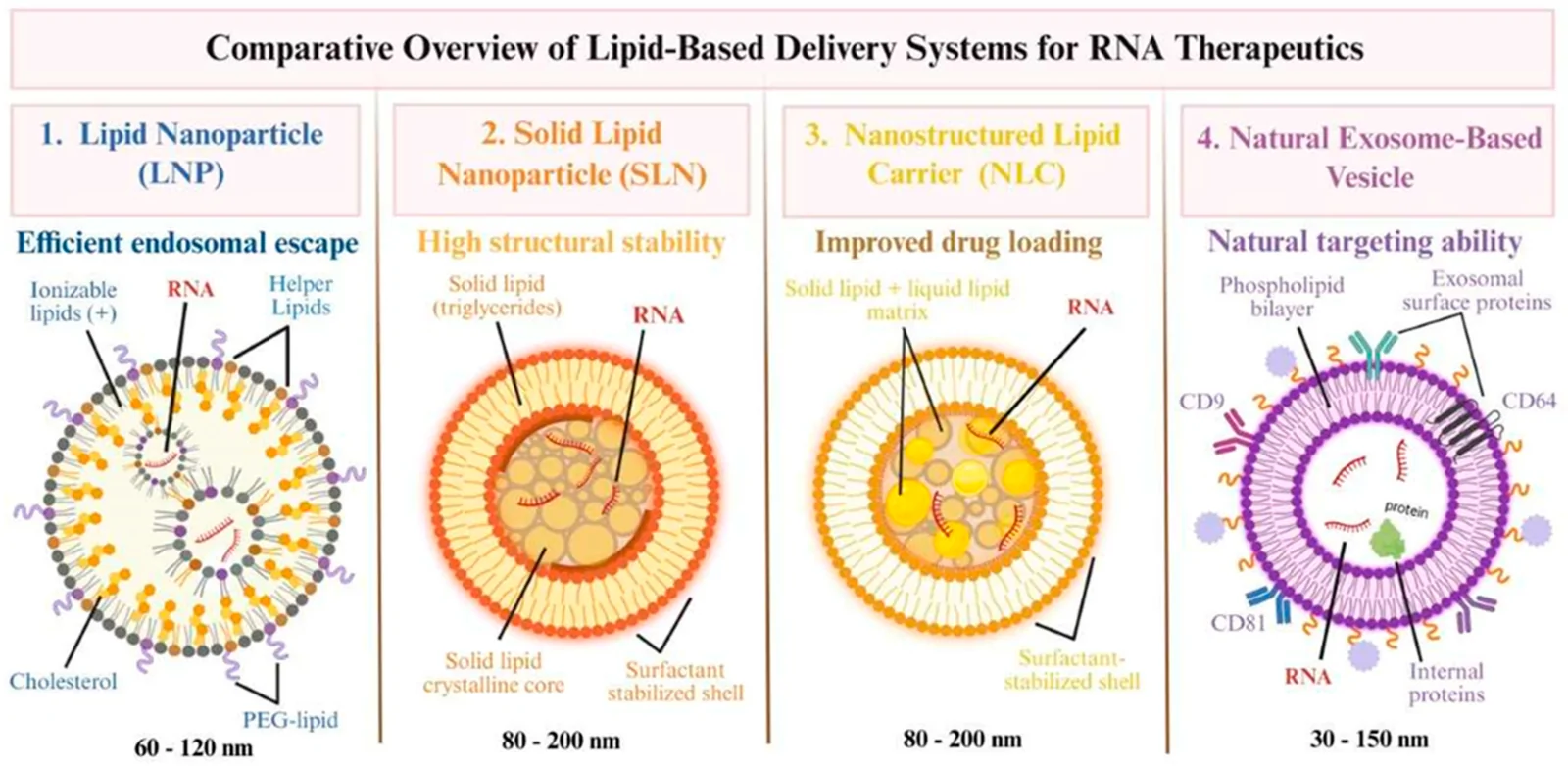
**A Schematic representation of nanocarriers for RNA delivery.** The structural and quantitative features of four lipid-mediated RNA delivery systems: **(1) Lipid nanoparticles (LNPs)** consist of ionizable lipids, helper lipids, cholesterol, and PEG-lipids containing the RNA payload; they typically exhibit a particle size range of 60–120 nm. **(2) Solid lipid nanoparticles (SLNs)** have an ordered solid lipid core protected by surfactants; they feature a size range of 80–200 nm. **(3) Nanostructured lipid carriers (NLCs**) have a complex combination of solid and liquid lipids to enable high cargo capacity; they present a size range of 80–200 nm. **(4) Natural exosome vesicles** are naturally occurring vesicles that facilitate cell–cell interactions and targeted delivery. Featuring a phospholipid bilayer with membrane proteins and endogenous biomolecules, they possess a biological size range of 30–150 nm. Created in BioRender. Abualsaud, N. (2026) https://BioRender.com/22vsgfl. accessed on 7 July 2026.

Nevertheless, it is worth highlighting that the design of these vehicles requires balancing interconnected variables, including delivery efficacy, immunogenicity, biocompatibility, scalability, and formulation stability [27]. Therefore, various types of nanocarrier platforms have emerged, each with a unique composition and structural configuration and limitations. While all RNA nanocarriers share the common goal of protecting and delivering nucleic acids, they differ fundamentally in their structural architecture, mechanisms of intracellular RNA release, physicochemical properties, and clinical maturity. These distinctions largely determine their transfection efficiency, safety profile, and therapeutic applications [28]. To address these requirements, various delivery systems have been designed; the primary ones among these are lipid-based architectures [29], polymeric polyplex structures [30], as well as emerging bioinspired systems such as exosome-mimicking carriers [31]. Among the several types of nanocarrier techniques available today for avoiding the obstacles in the delivery of RNA, lipid-based systems have achieved the highest levels of clinical maturity with their LNPs, solid lipid nanoparticles (SLNs), and nanostructured lipid carriers (NLCs) (Figure 1) [18,29]. All these structures share a unique composition that provides them with particular characteristics, which would be determined by the relevance of the RNA payload [32]. However, there is an array of both lipids and polymers under development for dealing with other difficulties involved in RNA delivery, such as challenges of stability, efficacy, and scalability [33]. Accordingly, the following sections discuss the principal RNA nanocarrier platforms, highlighting their structural characteristics, mechanisms of intracellular RNA delivery, advantages, limitations, and current stage of clinical development.

### 2.1. Lipid Nanoparticles (LNPs)

Currently, lipid nanoparticles are the gold standard as well as the most clinically advanced RNA delivery platform today. This is a finding fundamentally corroborated in the clinic with the worldwide success of SARS-CoV-2 mRNA vaccines [34]. Furthermore, besides their use in vaccines, the potential of LNPs as a therapeutic modality has been well established before. For instance, the FDA approved a system that uses LNPs in delivering siRNA in the liver for therapeutic purposes [17].

The clinical performance of LNPs depends more on proper structural organization rather than on mere stoichiometry [18]. Therefore, LNPs depend on an advanced quaternary structure to be effective. In the core, an ionizable cationic lipid enables the formation of the core structure. Besides other components, the core is stabilized using appropriate amounts of helper phospholipid, typically 1,2-distearoyl-sn-glycero-3-phosphocholine (DSPC) [27]. Furthermore, to adjust bilayer fluidity and rigidity, cholesterol is added. The final component, PEGylated lipid, is incorporated to provide a “stealth” surface, effectively shielding the particle from rapid systemic clearance [35].

Within this specific arrangement, the ionizable lipid functions as the primary operational engine. It is engineered for a dual existence: maintaining near-total electrical neutrality at a systemic pH ~7.4 to bypass nonspecific cellular interactions and, importantly, to suppress off-target toxicity while in the bloodstream. An essential biochemical transition is initiated once the particle is sequestered within the endocytic pathway. With further maturation and acidification (down to pH 5.5–6.0) of the endosome, the tertiary amine groups of the ionizable lipid start becoming more protonated [17]. The formation of the positive charge creates an electrostatic interaction with the negatively charged lipids in the endosomal membrane (phosphatidylserine), leading to the formation of a non-bilayer hexagonal (*H*_II_) phase. This phase transition disrupts the endosomal membrane, thus creating a temporary pore. Therefore, this causes the “charge-to-release” process, which results in the dissociation of the LNP structure, releasing the mRNA cargo from the lipid shell that can diffuse freely into the cytoplasm [36,37].

Protonated lipids bind to anionic lipids in the endosomal membrane, causing membrane disruption and release of RNA cargo into the cytoplasm due to membrane instability [38]. Cholesterol gives structural support and stability, and PEG-lipids can provide “stealth” by blocking opsonization and its elimination by mononuclear phagocytes [39].

Moreover, LNPs deliver negatively charged molecules such as mRNA and siRNA particularly efficiently, since they exert excellent transfection capacity and high encapsulation efficiency [40]. RNA encapsulation can be widely accomplished by a rapid-mixing approach, specifically microfluidic-assisted nanoprecipitation, which has high encapsulation efficiency and can be mass-produced quickly with narrow particle size distributions [41].

Even though LNPs play a crucial part in the efficiency of COVID vaccines, these carriers have several limitations that are clinical and logistical in nature. The leading among them is that they have poor thermal stability [42]. Furthermore, a continuous and stringent cold chain needs to be followed in order to protect the genetic payload from degradation. Besides issues related to storage, the addition of PEGylated molecules to increase colloidal stability and the period of circulation has been found to work both ways. It has been observed that such molecules tend to cause anti-PEG antibodies, which could lead to faster blood clearance of the drugs [43].

### 2.2. Solid Lipid Nanoparticles (SLNs)

SLNs comprise lipid-based drug delivery systems containing solid lipid cores [44]. The lipid materials used in the nanoparticles retain their solid-state and crystalline characteristics in both room and physiological temperature conditions [45]. During the formulation, to maintain the integrity of the internal solid lipid structure, surfactants are used as essential stabilizing agents [46]. Consequently, this makes the physical structure rigid, offering stability to the lipids against aggregation when in storage. Moreover, SLNs are commonly functionalized with biocompatible and degradable lipids for promising biocompatibility [47].

For the purpose of delivering RNA, SLNs are typically loaded with cationic lipids and polymeric compounds that enable electrostatic interactions between the RNA and the nanoparticles [4]. By acting as a physical barrier, the solid lipid provides partial protection against enzymatic degradation of the RNA and facilitates its entrapment in the core of the nanoparticles [48]. However, the release mechanisms of RNA molecules from SLNs differ from those of LNPs. While the LNPs are triggered by rapid pH-dependent environmental changes, the SLNs largely depend on the process of slow erosion of the solid crystalline lipid matrix as well as the diffusion of the entrapped molecule through the pores created during the erosion and enzymatic degradation. Although this results in the gradual, prolonged release of the cargo, the high order and rigidity of the solid lipid matrix significantly reduce the capacity for accommodating large nucleic acids. This limits large mRNA molecules from being loaded efficiently into SLNs [18]. In addition, the storage phase transition may cause the compression of the matrix, which leads to the premature release of the entrapped RNA payload [35].

### 2.3. Nanostructured Lipid Carriers (NLCs)

Nanostructured lipid carriers (NLCs) are developed as second-generation lipid-based systems, which are supposed to address the issues faced by SLNs [32]. The incorporation of solid and liquid lipids in NLCs provides an amorphous matrix with high accessibility space [29]. This structural alteration enhances RNA loading capability, stability during storage, and prevents RNA leakage during the storage period [40].

The liquid lipid component breaks down the crystal packing structure and introduces “pockets” for RNA molecules [49]. The localized disruption in the crystal structure causes a unique delivery system characterized by high uniformity. In contrast to the slow surface degradation of SLNs, the fluid spaces created in the NLC matrix enable easy and uninterrupted diffusion of the RNA payload following its internalization, preventing unexpected and rapid cargo expulsion. The particular structural arrangement of the matrix allows it to better tolerate polymorphic transformation during storage, thus enhancing its physical stability and minimizing leakage [45]. On the other hand, the tightly arranged crystal structure of SLNs restricts internal space, which means that any polymorph change will lead to cargo expulsion from the matrix [42].

### 2.4. Polymer-Based Nanocarriers

The polymer-based nanocarriers rely on the assembly and formation of electrostatic complexes, also referred to as polyplexes, through interactions between cationic or ionizable polymers and negatively charged RNA molecules. Furthermore, conventional polymer systems derived from polyethyleneimine (PEI), chitosan, and biodegradable polyesters have shown potential for RNA delivery owing to their high nucleic acid binding affinity and tunable physicochemical properties [33]. These demonstrated platforms can be readily modified by altering molecular weight, charge density, hydrophobicity, and degradation kinetics to maximize their delivery performance [30]. Consequently, the polymeric nanocarriers effectively protect RNA from enzymatic degradation and promote cellular uptake via electrostatic interactions with negatively charged cell membranes.

To overcome the limitations associated with conventional polyplexes, considerable efforts have focused on the development of next-generation polymeric carriers with improved intracellular delivery properties. Among these, the development of charge-altering releasable transporters (CARTs) has emerged as a promising platform that initially condenses RNA through electrostatic interactions before undergoing chemical rearrangement into neutral or weakly anionic by-products following cellular internalization, thereby facilitating efficient cytosolic RNA release [50]. Likewise, amphiphilic xenopeptides, which contain alternating hydrophobic and cationic domains, promote efficient RNA encapsulation and effective membrane interaction while maintaining low toxicity [51]. Similarly, Janus dendrimers precisely self-assemble into amphiphilic structures, thereby optimizing RNA encapsulation, controlled self-assembly, and targeted intracellular delivery [52]. Collectively, these next-generation polymeric systems illustrate how rational molecular design can simultaneously improve transfection efficiency, intracellular RNA release, and biocompatibility while overcoming several limitations of conventional polycationic carriers.

A major challenge associated with conventional polycationic carriers is the assembly–disassembly paradox. Strong electrostatic interactions are required to protect polyanionic RNA during extracellular transport. However, these same interactions may hinder intracellular RNA release after endosomal escape [53].

Failure of the polyplex to dissociate efficiently results in persistent sequestration of RNA within the carrier, thereby preventing ribosomal access and reducing protein translation. Moreover, some conventional polymers use the well-known mechanism of the “proton sponge,” which is based on the ability of the polymer to buffer pH value, causing the massive inflow of protons and chloride ions into the endosome, leading to its physical disruption [54]. Although this mechanism has been widely proposed to contribute to intracellular delivery, its precise contribution remains under investigation and may vary among different polymer systems. Nevertheless, the high cationic charge density is associated with cytotoxicity, nonspecific cellular interactions, and undesirable immune response [33]. Additionally, the batch-to-batch variability and difficulties regarding large-scale reproducible synthesis present barriers to regulatory approval [55]. Generally, polymers as nanocarriers provide excellent molecular customization and keep evolving with dynamic stimuli-responsive chemistry, thus presenting promising alternatives to lipid-based approaches in the future.

### 2.5. Hybrid and Exosome-like Nanocarriers

Biomimetic nanocarriers, including exosome-like systems, are inspired by naturally occurring extracellular vesicles. They possess intrinsic immune evasion capabilities and a precise targeting profile [31]. It is worth mentioning that the implementation of these emerging drug delivery strategies can be considered promising, as the use of exosome-like biomimetic nanoparticles combines the benefits of synthetic nanomaterial design and natural membrane materials, which will mitigate the rapid clearance and immune recognition [56]. In addition, the engineered hybrid nanomaterial includes polymer cores and lipid shells, thus allowing for structural stability and increased biocompatibility. The use of exosome-like particles provides increased RNA protection from the external environment and enhanced endosomal escape in comparison with other carriers [57]. The intracellular delivery of the RNA using exosome-like platforms is purely biological. After entering the cell, these platforms use the natural biological approach of membrane fusion or hemifusion to integrate with the internal membranes of the cell and transport the functional RNA cargo directly to the cytosol [47].

However, despite their biological features, the major concern is how such nanoparticles could be manufactured in large quantities and loaded with RNA efficiently while being classified from a regulatory point of view [31]. Despite the remarkable clinical success of the lipid-based nanocarriers, especially mRNA vaccines, no universal carrier has been created so far.

The nanocarriers based on lipids exhibit comparatively better endosomal escape than other delivery platforms; however, certain limitations have been seen, such as hepatic tropism, PEG immunogenicity, and the requirement of cold-chain preservation [17,42]. Furthermore, SLN and NLC have offered better physical and thermal stability; however, they can be subject to structural limitations due to lipid polymorphism [18]. Additionally, polymeric nanocarriers show great modulatory and degradation control properties, although toxicity and batch-to-batch variations are issues with the polymeric nanocarriers [30]. The emergence of the new generation of hybrid and bioinspired nanocarriers has been proposed, which would be a combination of higher structural stability and enhanced immune evasion capability. However, large-scale production continues to present challenges, and the classification of their consistency remains debatable for clinical use [58]. A comparative summary of the major nanocarrier platforms used for RNA delivery is presented in Table 1.

## 3. Formulation Strategies for RNA-Loaded Nanocarriers

The composition and synthesis of RNA-loaded lipid nanoparticles are critical for delivery efficiency, safety, manufacturability, and clinical translational applications [59]. Their biological performance is governed not only by composition, but also by how formulation controls nanoparticle assembly, RNA complexation, internal organization, colloidal stability, and batch-to-batch reproducibility [37]. The LNP landscape has evolved from simple RNA encapsulation to a systems-level design framework. Present strategies include the co-optimization of chemical ionizable lipids, sterols, and helper lipid molar ratios, the biophysical nitrogen-to-phosphate ratio, microfluidic mixing, and post-formulation variables. This integrated approach is essential to synchronize cargo retention with organ-selective biodistribution and endosomal escape, ultimately maximizing the therapeutic index while minimizing immunogenicity [60,61,62]. RNA-LNPs generally comprise four principal components: an ionizable lipid, a helper phospholipid, cholesterol or a cholesterol analog, and a PEG-lipid, typically assembled by rapid mixing of an ethanolic lipid phase with an acidic aqueous RNA phase (Table 2) [63]. Formulation development can be a mechanistic determinant of vaccine efficacy, not merely a manufacturing step. Even with identical profiles (e.g., size, PDI, and encapsulation), structural internal lipid organization or PEG kinetics can dictate biological fate, in governing lymph node trafficking and the resulting immune response.

The primary function of ionizable lipids is to mediate electrostatic RNA complexation during acidic self-assembly and facilitate endosomal escape via membrane destabilization upon cellular uptake [66]. These lipids are designed to stay neutral at physiological pH, which reduces nonspecific protein adsorption, opsonization, and systemic toxicity compared to permanently cationic counterparts. After the internalization of LNPs, protonation within acidic endosomes triggers interactions with anionic endosomal membranes, supporting membrane destabilization and resulting in cytosolic RNA release [64]. This design facilitates the development of immunocompatible RNA-LNPs. By incorporating near-neutral ionizable lipids with the biocompatible structural main lipids and PEGylated lipids, the carriers can preserve intracellular delivery with minimum immunogenicity or innate immune activation [81]. While a pKa between 6.0 and 6.8 is necessary for pH-responsive delivery, it is insufficient to predict transfection potency. Recent structure-function analyses reveal that molecular architecture, including headgroup chemistry, linker biodegradability, and hydrophobic tail branching or unsaturation, serves as a primary determinant of membrane-fusogenicity [82]. These features dictate the lipid’s ability to undergo the lamellar-to-inverted hexagonal phase transition required for cytosolic RNA release [82]. In a comparative study, SM-102 and ALC-0315 both had over 95% encapsulation efficiency, but SM-102 LNPs created smaller particles and yielded about 60% more luciferase expression in mice [83]. Mechanistic studies show that inverted hexagonal phases in LNP nanostructure can boost endosomal escape and transfection, indicating that small compositional differences greatly impact potency beyond standard physicochemical measures [65,84]. Together with recent evidence linking SM-102’s superior activity to inverted hexagonal internal phases that enhance endosomal escape, this highlights that subtle compositional differences can strongly influence LNP potency beyond conventional physicochemical metrics [65].

Helper excipients are active determinants of RNA-LNP structure, stability, potency, and immunocompatibility rather than passive structural additives [40]. Phospholipids, for example, distearoylphosphatidylcholine (DSPC) and dioleoylphosphatidylethanolamine (DOPE), can function as primary structural determinants that dictate the molecular packing density, interfacial tension, and membrane fusion potential of the lipid bilayers. Alongside this, cholesterol can serve as a critical regulatory sterol, modulating the mechanical stiffness, thermodynamic phase transitions, and the kinetic pathways of membrane fusion [67,68]. PEG-lipids further control particle size, steric stabilization, protein corona formation, and circulation behavior by generating a hydrophilic surface that limits aggregation and reduces recognition by the mononuclear phagocyte system, thereby helping to minimize immune clearance [71]. The clinically validated mRNA-LNP systems of Pfizer-BioNTech and Moderna COVID-19 vaccines demonstrate how the rational selection of the excipient can support both efficient delivery and reduced immunogenicity by the combination of ionizable lipids, phospholipids, cholesterol, and PEG-lipids. Crucially, variations in the phospholipid choice, the selection and acyl chain length of PEG-lipids, or the concentration and structural analogs of sterols can fundamentally influence the tissue tropism, dictate intracellular trafficking pathways, optimize transfection potency, and affect the scalability and reproducibility of the manufacturing process [67]. Accordingly, formulations with similarly favorable bulk properties, including sub-100 nm particle size, low polydispersity, near-neutral zeta potential, and high apparent encapsulation efficiency, may still exhibit substantially different in vivo performance [85]. Consistent with this, SM-102- and ALC-0315-based formulations can outperform systems incorporating DLin-MC3-DMA, DODAP, or DOTAP in mouse mRNA delivery, whereas DSPE-PEG2000 may reduce expression relative to ALC-0159 or DMG-PEG2000, despite comparable measured physicochemical characteristics [69,70].

A complementary strategy for reducing immunogenicity resides in the RNA cargo itself. The incorporation of N1-methyl-pseudouridine into synthetic mRNA has become a defining feature of modern mRNA-LNP systems because it enhances molecular stability, improves translational efficiency, and attenuates innate immune sensing [72]. The m1Ψ-modified mRNA reduces recognition by endosomal Toll-like receptors, including TLR3 and TLR7/8, as well as cytosolic RNA sensors such as RIG-I and MDA5, thereby limiting type I interferon signaling and pro-inflammatory cytokine production that would otherwise suppress translation and increase reactogenicity [73]. In parallel, m1Ψ improves mRNA structural stability, ribosomal decoding, and intracellular persistence, resulting in stronger and more sustained protein expression [74]. Thus, the favorable immunological profile of contemporary mRNA-LNP systems should be viewed as the combined consequence of carrier engineering and nucleoside modification rather than lipid optimization [74].

**Figure 2:**
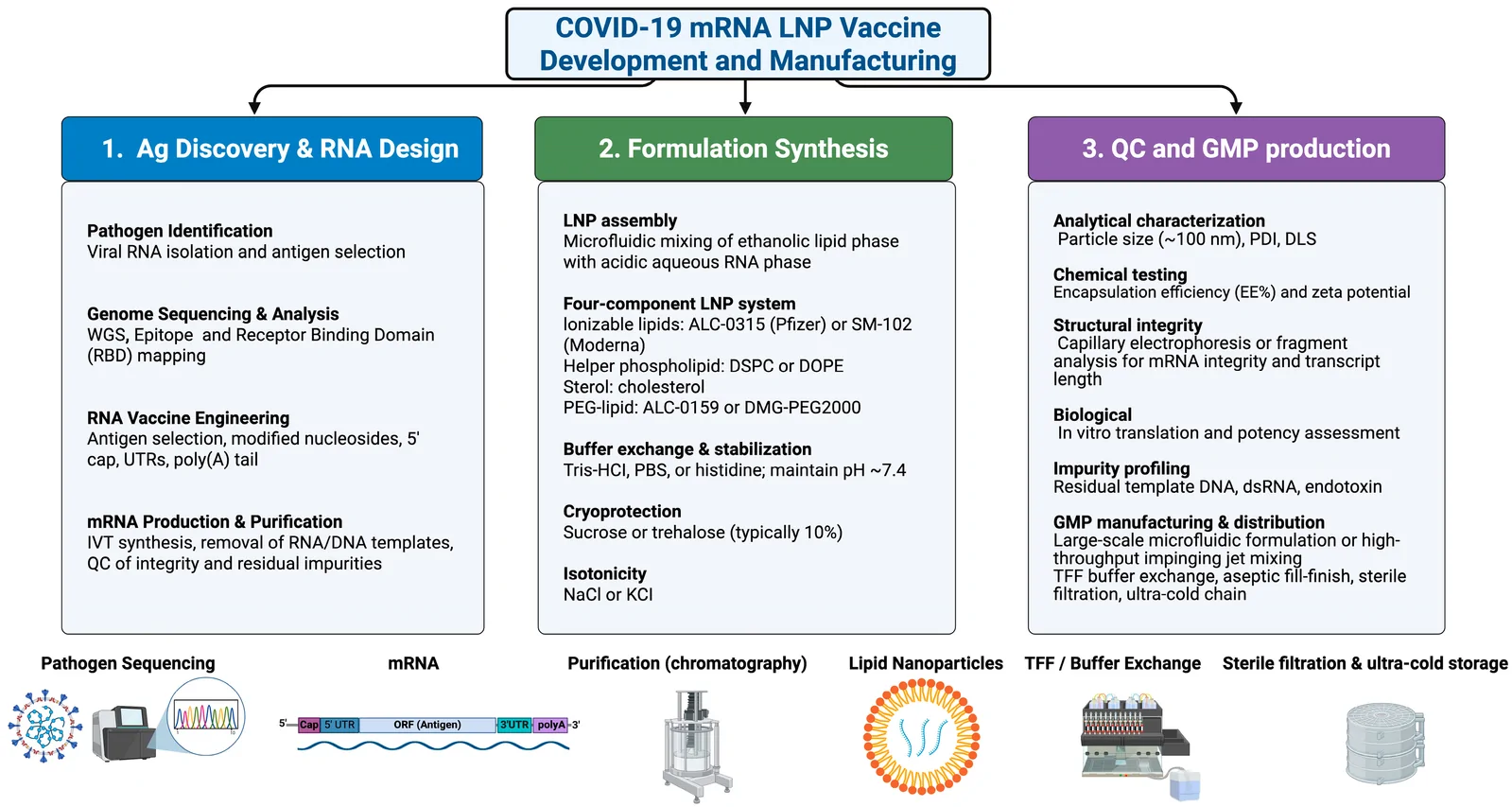
COVID-19 mRNA-LNP vaccine development and manufacturing. The figure outlines the integrated workflow for COVID-19 mRNA-LNP vaccine development, from antigen discovery and RNA design to formulation synthesis and GMP production. After pathogen identification, genome sequencing, RNA engineering, and mRNA production, lipid nanoparticles are assembled by microfluidic mixing of an ethanolic lipid phase with an acidic aqueous RNA phase. The formulation is then stabilized by buffer exchange, cryoprotectants, and isotonicity adjustment, followed by analytical characterization, structural and chemical quality testing, biological potency assessment, impurity profiling, tangential flow filtration, sterile filtration, and ultra-cold storage. The bottom panel depicts the simplified stepwise sequence from pathogen sequencing and mRNA generation to purification, LNP formation, buffer exchange, and final storage. Created with BioRender.com. Abbreviations: Ag, antigen; RNA, ribonucleic acid; mRNA, messenger RNA; LNP, lipid nanoparticle; IVT, in vitro transcription; WGS, whole genome sequencing; RBD, receptor binding domain; UTR, untranslated region; DSPC, 1,2-distearoyl-sn-glycero-3-phosphocholine; DOPE, 1,2-dioleoyl-sn-glycero-3-phosphoethanolamine; PEG, polyethylene glycol; TFF, tangential flow filtration; QC, quality control; GMP, good manufacturing practice; PDI, polydispersity index; DLS, dynamic light scattering; EE, encapsulation efficiency. Alradwan, I. (2026) https://BioRender.com/m8lnv9j, accessed on 6 July 2026.

Formulation processing is a major determinant of RNA-LNP quality and function, and the overall development and manufacturing workflow of mRNA-LNP vaccines is summarized in Figure 2. Microfluidic mixing has emerged as a preferred platform for laboratory-scale and translational LNP production because it enables rapid and reproducible nanoparticle self-assembly through controlled solvent exchange under laminar flow conditions [86]. While microfluidic mixing technologies can provide more precise control over formulation parameters at laboratory scale and pilot development, the high-throughput impinging jet mixers (IJM), on the other hand, can utilize the turbulent flow regimes to achieve the rapid mixing kinetics required for large-scale good manufacturing practice (GMP) for large-scale production [86]. In this process, lipids are dissolved in ethanol and are combined with RNA in an acidic aqueous buffer, usually citrate or acetate, to allow for nanoparticle self-assembly within milliseconds, in which the protonated ionizable lipids associate electrostatically with RNA [77]. Post-formulation, dilution, and buffer exchange procedures can facilitate the removal of residual ethanol, the return of physicochemical equilibrium at near-neutral pH, and the final transition of the particles into an isotonic medium [77]. Microfluidic process parameters, including total flow rate, flow rate ratio, and mixer architecture, can substantially influence LNP size, size distribution, morphology, and biological performance, with these effects becoming particularly important for larger or more complex cargos, such as Cas9 mRNA and guide RNA co-formulations [76,87]. Although encapsulation efficiency is an important indicator of dose accuracy and process yield, it should not be interpreted in isolation, as RiboGreen-based assays may overestimate performance by failing to distinguish properly encapsulated RNA from RNA associated with incomplete particles or lost during downstream processing [34]. Encapsulation efficiency should not be interpreted solely from conventional RiboGreen assay results; RNase protection assays should also be considered to distinguish truly protected, internalized RNA from externally associated or partially exposed RNA and to provide a more functionally relevant assessment of formulation quality [88]. Consequently, encapsulation efficiency (EE%) must be assessed alongside hydrodynamic diameter, PDI, zeta potential, and structural morphology. A thorough evaluation of formulation quality also necessitates the validation of RNA primary structure integrity, transfection efficacy, and long-term storage stability [89].

There is a growing agreement that buffer systems and excipients can function as active modulators of RNA-LNP in thermodynamic stability and biological efficacy, working beyond their traditional assigned roles as inert or passive delivery vehicles [80]. While acidic conditions are required for initial self-assembly, the final formulation must achieve physicochemical equilibrium at neutral pH and be optimized for ionic strength and be resistant to hydrolytic degradation, oxidative insults, colloidal aggregation, and cryogenic (freeze–thaw) stress [60,90,91]. In this context, buffers and cryoprotectants such as Tris, phosphate, histidine, and sucrose can play an important role in preserving particle integrity, shelf stability, and stress tolerance, while indirectly influencing immunocompatibility by limiting the structural degradation that could expose reactive or immunostimulatory surfaces [79]. Buffer composition can also influence biological performance. For instance, high citrate concentrations may reduce cellular uptake and transfection efficiency even when physicochemical properties appear to be similar [78]. Meanwhile, mildly acidic histidine-based buffers have been reported to enhance room temperature stability in certain siRNA LNP formulations by limiting the oxidation and RNA-lipid adduct formation [78]. Also, cryoprotectants such as sucrose can further enhance the freeze–thaw stability but may modestly increase particle size [79]. Esterified fatty acids may also be incorporated to tighten membrane packing, reduce cargo leakage, and further improve formulation stability and immunocompatibility [67].

## 4. Stability Engineering of RNA Nanocarriers

In addition to the formulation strategies, the stability of RNA-loaded nanocarriers must be carefully engineered. Improving the robustness of these platforms is a complex challenge that involves addressing both the chemical vulnerability of the RNA cargo and the physical fragility of the lipid or polymer carrier. Stability remains a key bottleneck in RNA vaccine development. Encompassing chemical degradation of RNA, physical instability of the nanocarrier, and loss of functional potency during storage, transport, and handling could affect stability. Effective stability engineering requires integrating molecular RNA design, nanocarrier formulation optimization, and process-level control strategies to maintain vaccine potency and safety throughout its lifecycle. In this section of the review, we focus on chemical stability and degradation mechanisms, physical stability and aggregation prevention, lyophilization, and thermostability enhancement.

### 4.1. Chemical Stability and Degradation Mechanisms

**Figure 3:**
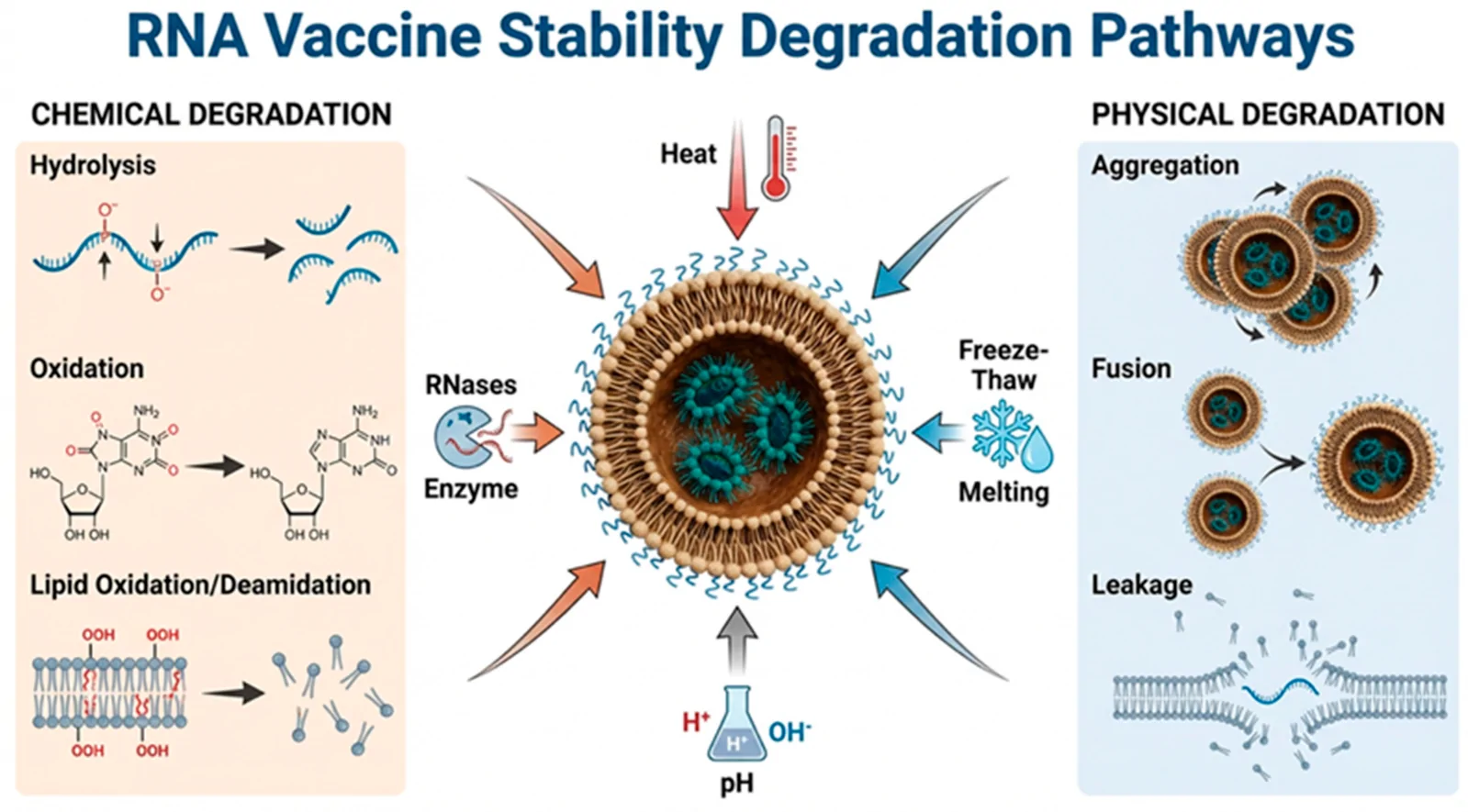
RNA vaccine stability degradation pathways. This three-part figure illustrates the major mechanisms contributing to instability in RNA-lipid nanoparticle (LNP) systems. (**Left**) Chemical degradation pathways, including hydrolysis driven by the 2′-hydroxyl group of RNA, oxidative damage to nucleobases, and lipid oxidation/deamination processes that can compromise both RNA integrity and carrier stability. (**Center**) Environmental and enzymatic stressors affecting LNP-encapsulated RNA, such as temperature fluctuations (heat), pH extremes, freeze–thaw cycles, and RNase-mediated degradation, highlighting the partial protective role of LNP encapsulation. (**Right**) Physical degradation mechanisms of LNPs, including aggregation, particle fusion, and leakage of RNA cargo, which collectively reduce delivery efficiency and stability. Together, these interconnected pathways highlight the need for integrated stabilization strategies across chemical, environmental, and structural levels.

RNA molecules are intrinsically unstable due to the presence of the 2′-hydroxyl group on the ribose backbone. This promotes base-catalyzed hydrolysis via intramolecular transesterification, making RNA more labile than DNA under physiological and storage conditions. This degradation pathway is influenced by many factors. These factors include pH, temperature, and water activity, with alkaline conditions accelerating strand cleavage [92]. In addition to hydrolysis, enzymatic degradation by RNases remains a major challenge, as even trace contamination during manufacturing or handling can rapidly degrade RNA (Figure 3). Encapsulation within LNPs provides partial protection but does not fully prevent exposure to residual water and reactive species [93]. These degradation mechanisms are illustrated in Figure 3.

Oxidative degradation is another major pathway reported by [94], particularly in lipid-based systems. Lipid peroxidation leads to the formation of ROS, which can damage nucleobases and ultimately impair translational efficiency. The link between lipid oxidation and RNA degradation highlights the need for stabilization strategies targeting both the cargo and the carrier. Formulation composition, including buffer selection and the ionic environment, plays a major role in RNA stability. This effect becomes especially noticeable under stress conditions such as freezing and drying [92]. To address these challenges, chemical stabilization strategies concentrate on nucleotide variations, buffer optimization, and reduction in water activity. Modified nucleosides such as N1- methylpseudouridine ameliorate translational effectiveness and reduce immune activation. On the other hand, optimized buffers maintain pH conditions that minimize hydrolysis [93]. Likewise, drying technologies such as lyophilization reduce water-mediated degradation. These technologies represent a critical ground between chemical and process-level stability engineering [94]. To really understand RNA nanocarrier stability, it helps to look at it as a multi-scale problem that includes chemical stability, structural changes, and how long the RNA remains functional, rather than focusing solely on molecular degradation. Crucial stability challenges and corresponding engineering strategies for RNA vaccine nanocarriers are epitomized in Table 3.

### 4.2. Physical Stability and Aggregation Prevention

Beyond chemical degradation, RNA nanocarriers are also prone to physical instability, which can compromise delivery efficiency even when RNA integrity is preserved. LNPs are colloidal systems stabilized by a balance of electrostatic and steric forces. When this balance is disrupted, it can lead to aggregation, fusion, and changes in particle size distribution, ultimately affecting biodistribution and cellular uptake [92,93]. These physical instability mechanisms, including aggregation, fusion, and leakage, are illustrated in Figure 3. Aggregation is a major issue during storage and handling, where environmental stressors such as temperature fluctuations, mechanical agitation, and freeze–thaw cycles can destabilize the nanoparticle structure. Freeze–thaw stress induces ice crystal formation, which concentrates solutes and brings particles into proximity, promoting irreversible aggregation [95]. Similarly, lipid phase transitions at elevated temperatures can disrupt membrane integrity and cause cargo leakage.

LNP composition plays an important role in determining physical stability. Ionizable lipids, which are essential for RNA encapsulation and endosomal escape, also impact stability during storage and processing. The success of lyophilization and long-term storage depends strongly on the specific ionizable lipid used [99]. Cholesterol contributes to membrane rigidity, while PEG-lipids give steric stabilization, reducing particle–particle interactions and aggregation [100]. To alleviate physical instability, cryoprotectants such as sucrose and trehalose are commonly used. These excipients form a protective matrix that helps separate nanoparticles and stabilizes lipid bilayers during freezing and drying [95]. More advanced strategies include zwitterionic surface modifications to enhance colloidal stability and reduce immunogenicity [101], as well as crosslinking approaches that improve structural integrity and delivery effectiveness [102]. Overall, these strategies help preserve nanoparticle structure across a range of stress conditions.

### 4.3. Lyophilization Approaches

**Figure 4:**
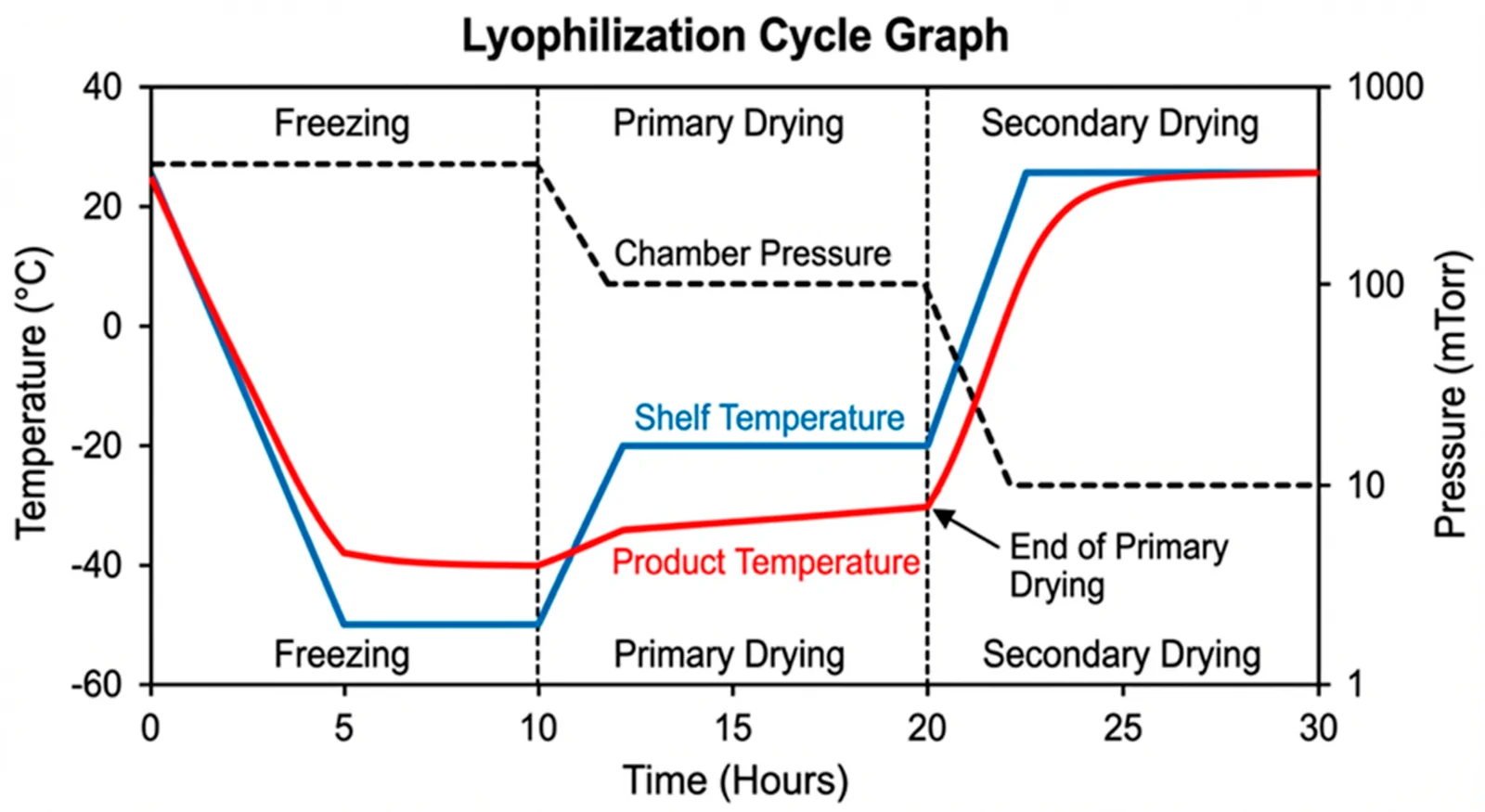
Schematic representation of a lyophilization cycle showing changes in shelf temperature, product temperature, and chamber pressure over time. The x-axis represents time, while the y-axis represents temperature and pressure. The blue line indicates shelf temperature, the red line indicates product temperature, and the dashed black line indicates chamber pressure. The cycle is divided into three stages: Freezing, where temperature decreases to approximately −50 °C; primary drying, where shelf temperature gradually increases to around −20 °C while chamber pressure drops significantly; and secondary drying, where temperature rises to approximately +25 °C under low-pressure conditions. The point at which all ice has sublimated is marked as the End of Primary Drying.

Lyophilization (freeze-drying) has emerged as a cornerstone technology for stabilizing RNA nanocarriers, enabling long-term storage by removing water and immobilizing both RNA and lipid components. The process involves three key stages: freezing, primary drying, and secondary drying. These stages and their associated temperature and pressure profiles are shown in Figure 4. Each stage must be carefully optimized to prevent structural damage and preserve biological activity [96]. During freezing, ice crystal formation can induce mechanical stress and solute concentration effects that destabilize LNPs. Controlled freezing rates and annealing steps are thus used to regulate ice crystal size and distribution [97]. Primary drying removes ice via sublimation under reduced pressure, while secondary drying eliminates residual bound water to achieve moisture levels generally below 1–3%, which is critical for long-term chemical stability [98].

A crucial determinant of successful lyophilization is the selection of cryoprotectants, particularly disaccharides such as sucrose and trehalose. These molecules stabilize both RNA and lipid structures via hydrogen bonding and vitrification, resulting in the formation of a glassy matrix that prevents aggregation and degradation [95]. Recent work shows that mixed cryoprotectant systems can improve process robustness by increasing collapse temperature and enabling faster drying cycles [103]. As shown in Table 3, cryoprotectants and optimized freeze-dry cycles are key approaches for maintaining RNA and LNP stability during lyophilization.

A notable innovation is internal trehalose co-loading, where trehalose is encapsulated within LNPs alongside mRNA rather than added externally. This approach provides more direct stabilization of the RNA cargo. Also, shown to enhance both stability and in vivo efficacy by reducing oxidative stress [89]. Alternative processing methods such as continuous freeze-drying offer improved scalability and manufacturing efficiency compared to traditional batch processes [103]. Optimized lyophilization protocols show that mRNA-LNPs can be dried and reconstituted with minimal changes in particle size, encapsulation efficiency, and biological activity. Several studies [99,104] reported marking a significant step toward practical deployment.

### 4.4. Thermostability Enhancement

Improving thermostability is essential for overcoming the limitations of cold-chain logistics associated with RNA vaccines. Traditionally, mRNA-LNP formulations have required storage at ultra-low temperatures (−70 °C to −80 °C). That posing significant challenges for global distribution. Advances in stability engineering have enabled substantial improvements in storage conditions, particularly through lyophilization and formulation optimization. Lyophilized mRNA-LNP vaccines have demonstrated long-term stability at refrigerated temperatures (2–8 °C). Studies have reported maintained physicochemical properties and immunogenicity after up to 12 months of storage [97,100]. The temperature and pressure changes during the lyophilization cycle are illustrated in Figure 4. Highlighting conditions critical for maintaining RNA integrity. In some cases, formulations also show short-term stability at room temperature, allowing greater flexibility during transport and administration [97].

Emerging RNA platforms offer additional advantages for thermostability. saRNA formulated in NLCs has shown excellent stability, maintaining efficacy for several months at room temperature due to its self-replicating nature and robust lipid matrix [105,106]. Likewise, circRNA, which lacks free ends susceptible to exonuclease degradation, exhibits enhanced intrinsic stability and has demonstrated sustained delivery and immune responses after lyophilization [107].

These advances emphasize that thermostability is not solely dependent on drying processes. It is dependent on RNA design and nanocarrier architecture. The combination of optimized formulations, advanced excipients, and better RNA platforms is driving the development of next-generation vaccines that can be stored and distributed without strict cold-chain conditions. Stability engineering that integrates chemical, physical, and process-level approaches offers a pathway to better RNA vaccines suitable for practical deployment.

## 5. Biological Performance and Delivery Mechanisms

Nanovaccines have emerged as a potential vaccination strategy to induce cross-presentation by DCs and generate robust CD8+ T cell activation, and to overcome the limitations associated with conventional peptide-based vaccination approaches [108]. Nanovaccines have been shown to facilitate antigen presentation on MHC I and MHC II pathways, inducing both cellular and humoral immune responses, eventually supporting B cell activation and antibody production. In addition, nanovaccine adjuvants can contribute to DC activation through the engagement with pattern-recognition receptors on DCs, resulting in type I interferon expression and the release of antiviral cytokines. Due to these highly attractive properties, in addition to their enhanced delivery sufficiency, nanovaccines are currently considered as promising candidates that may replace conventional vaccine-based approaches for the induction of long-lasting immunity against diseases [108,109,110].

RNA drug delivery systems are crucial for targeted delivery; thus, different approaches have been developed to maximize the potential effect of RNA-based vaccines, including polymeric, lipid-polymeric, exosome-based, cell-based, and inorganic systems, with liposomes and LNPs being the greatest nanocarrier candidates in the clinic [111]. Liposomes are used due to their ability to protect therapeutic cargo from degradation and deliver drugs to target tissues and release them in a controlled manner. In addition, liposome structures carry both hydrophilic and hydrophobic drugs in their aqueous core within the lipid membrane [111].

The internal cavity of liposomes can accommodate long RNA molecules, where they remain protected until they reach target cells. Furthermore, liposomes are commonly used for targeted delivery because they can be easily modified by different techniques such as solvent injection, thin-film hydration, phase evaporation, freeze-drying, and microfluidics [112]. Liposomes can be administered by different routes, including oral, ocular, parenteral, pulmonary, and transdermal routes for diagnostic and therapeutic purposes [112].

Lipid nanoparticles are currently very attractive for targeted mRNA therapy. Their unique composition, which consists of ionizable lipids, cholesterol, PEG-lipids, and helper lipids, plays a major role in delivery because they become positively charged in the acidic environment of endosomes while they remain neutral at physiological PH. Consequently, this feature promotes endosomal destabilization and RNA escape into the cytosol [67]. Helper lipids maintain membrane structure and facilitate endosomal escape while enhancing particle stability. Cholesterol and PEG-lipids serve functions like those found in liposomal systems, improving nanoparticle stability and prolonging circulation time. The assembly of these lipid components forms a spherical nanoparticle, where ionizable lipids encapsulate and protect the RNA cargo [67].

Effective LNPs are typically 80 to 100 nm in size, neutrally charged, uniform, and highly efficient at RNA encapsulation. This technology has already been used in approved therapies such as Onpattro and mRNA COVID-19 vaccines, with modern microfluidic manufacturing enabling precise, scalable, and consistent production [111]. Despite their advantages, lipid-based RNA delivery approaches have some limitations. For example, this type of delivery system has the tendency to accumulate in the liver due to its interaction with apolipoprotein E (ApoE), facilitating the uptake by hepatocytes through low-density lipoproteins; thus, targeting the brain or bone marrow is challenging. Furthermore, even the most advanced lipid nanocarriers achieve very low endosomal escape rates, only around 1 to 2%, which minimizes delivery efficiency drastically [111]. However, high concentrations of PEG may lead to the development of anti-PEG antibodies, which may lead to rapid clearance of PEGylated particles from the blood and stimulate immune responses such as complement activation-related pseudoallergy [111,113].

Traditional lipid nanoparticles (LNPs) administered intravenously show a strong bias toward hepatic accumulation, limiting extrahepatic delivery of RNA therapeutics [1,114]. To address this limitation, the selective organ targeting (SORT) strategy was developed as a rational approach to reprogram LNP biodistribution by adding a fifth lipid component, termed a SORT molecule, to the conventional four-component LNP formulation [2,115]. Foundational studies showed that incorporating defined molar fractions of SORT molecules can alter in vivo organ targeting and enable extrahepatic delivery of mRNA and gene-editing systems [115,116]. This strategy has been shown to be generalizable across multiple LNP classes and supplementary lipid types, enabling delivery to the lung, liver, and spleen and supporting gene expression or CRISPR-Cas-based editing in therapeutically relevant cell types [116]. Composition-dependent targeting trends have been observed, with certain permanently cationic SORT lipids favoring lung delivery, whereas other auxiliary lipid classes can maintain hepatic delivery or redirect accumulation toward the spleen [116]. However, the mechanistic basis of SORT remains incompletely defined, and further study is needed to determine how the added lipid alters nanoparticle behavior in circulation and during tissue uptake [117]. Moreover, although SORT represents a major advance in organ-directed RNA delivery, its broader clinical translation will require improvements in tissue selectivity, formulation-dependent potency, and safety, particularly for lung-targeting systems that rely on permanently cationic components [118,119].

### RNA Delivery Systems and Immune Response

In vivo administration of mRNA vaccines has been shown to elicit host immune responses. For mRNA vaccines targeting cancer and infectious diseases, interactions with the immune system are fundamental to their effectiveness. Yet, it remains highly critical to assess the immunological effects in nonvaccine applications [120]. For cancer applications, designing tumor-specific antigens is crucial for immunogenicity; today, obtaining personalized antigens is showing promising outcomes [121]. The delivery system should induce sufficient immune responses against the targeted disease and prevent tumor relapse, which would benefit from the use of highly targeted nanoparticles designed to activate antigen-presenting cells. In infectious diseases, the delivered mRNA vaccine should be designed to activate the immune system without inducing inflammation, which could lead to reactogenicity [120]. The field has developed novel delivery approaches to avoid the delivered mRNA cargo from being recognized by the innate immune system. New carriers like ionizable lipids are now under investigation systematically to improve immunogenicity while reducing reactogenicity. A study by Pardi et al. demonstrated that using empty LNPs combined with a lipid mix and ionizable lipid adjuvanted the vaccine significantly [122]; however, another study showed that empty LNPs can activate human peripheral blood mononuclear cells (PBMCs) even in the absence of an antigen [123].

The endosomal escape ability of ionizable lipids, which disrupt intracellular vesicular membranes, may provoke inflammatory responses. In addition, accumulating data showed that LNPs can induce intracellular immune sensing pathways. Researchers have reported different pathways by different ionizable lipid formulations, and they are currently investigating their immunological mechanisms [124].

Determining the optimal mRNA vaccines for humans is still challenging; one of the main reasons is the differences in the immune responses following vaccination between human and animal models [125]. This issue might be resolved by utilizing systems vaccinology and the investigation of the immune reactions of carefully designed vaccines across species. Additional ex vivo techniques such as Modular iMmune In Vitro Construct (MIMC) that mimic the human immune system may predict the reaction and accelerate the discovery of potential vaccine formulations for different diseases [120].

Gene therapies, particularly mRNA delivery, provide a highly complex immune landscape. Some diseases that are caused by missing of a particular protein and treated by injecting the missing gene with multiple doses can cause an undesired result: inadequate central tolerance. In addition, systemic delivery of corticosteroids can lead to bone loss if they are given excessively [126,127]. While more research is needed for the mRNA case, preclinical and a number of clinical studies related to viral vector gene therapy suggested the ability of this type of vaccination to elicit antitransgene T cell responses against alpha-1 antitrypsin deficiency and cystic fibrosis [120].

The route of administration plays a critical role in determining the biodistribution, expression pattern, and the immune response of LNP-mRNA formulations. For example, Intravenous (i.v.) delivery often accumulation of the vaccine in the liver, enabling protein replacement therapy and antibody production; however, it has also been reported that i.v. injection can cause systemic immune responses and side effects, highlighting the need for an alternative and safer delivery approach [25]. Topical or local injections may provide a safer option because they allow targeted protein expression, including organs such as the heart, lungs, eyes, and the brain, with multiple approaches currently being evaluated in clinic trails. Well-established local injections, including intradermal and subcutaneous routes, are commonly used for vaccination, mainly due to the ability of the antigen-presenting cells in this tissue to uptake, process, and present the mRNA antigens on the cell surface and activate immune responses via lymph node trafficking [25]. Myocytes also induced robust immune responses following an intramuscular (i.m.) vaccination [128]. Additional strategies also include intranasal delivery and intertumoral injection to improve anticancer immune responses, and in utero delivery, which has demonstrated successful mRNA expression in fetal tissues in animal models. Additional strategies also include intranasal delivery and intertumoral injection to improve anticancer immune responses, and in utero delivery, which has demonstrated successful mRNA expression in fetal tissues in animal models.

## 6. Comparative Analysis of Nanocarrier Types

Nanocarrier functionality in terms of six functional parameters—encapsulation efficiency, cargo protection, endosomal escape, immunogenicity, transfection efficiency, and biodistribution—is vital for the translational success of RNA vaccines. The use of microfluidic systems and ionizable lipids (such as SM-102) results in a high mRNA encapsulation efficiency (EE) for LNPs of greater than 90%, which is significantly higher than that of modified PLGA nanoparticles (50–90%) or exosomes, which generally exhibit low loading capacity [129,130,131]. Although SLNs and NLCs can achieve 70–98% efficiency, NLCs are considered more effective than SLNs because their liquid lipid core creates an amorphous matrix that accommodates higher amounts of cargo [132,133]. To provide maximum protection against RNase degradation, LNPs and exosomes utilize dense lipid layers, whereas the polymeric matrix of PLGA remains vulnerable to hydrolysis in aqueous environments [131,134]. The most significant performance differences occur during endosomal escape; while the escape rate for commercially available LNPs is often limited to only 1–2%, certain exosomes can achieve efficiency rates more than 10 times higher (>20%) [87,124]. Exosomes utilize membrane fusion as a primary escape mechanism [135], whereas PLGA and SLNs typically show low escape efficiency without further surface modification [124,134]. Regarding immunogenicity, LNPs can stimulate Toll-like receptor 4 (TLR4), which may enhance vaccine efficacy but also carries a risk of causing inflammation [136], while exosomes possess natural biocompatibility and minimal immunogenicity [131,137]. The efficacy of LNPs to target the liver and spleen in vivo has been confirmed with transfection efficiencies exceeding 90%, primarily driven by ApoE binding and LDL-receptor-mediated endocytosis [87,136]. In contrast, exosomes offer high cell-type specificity and the ability to be engineered with ligands for precise targeting to other organs, such as the brain [131]. The systemic distribution of PLGA nanoparticles is size-dependent [138], while SLNs and NLCs are frequently utilized for localized or site-specific delivery, such as via the oral or topical routes [132,133].

The hierarchy of functional performance is now clear. Exosomes are best at endosomal escape and are the most biocompatible due to their natural origin [124,137]. LNPs are best at encapsulation efficiency and large-scale translational vaccine performance [97,139]. SLNs/NLCs are best at localization and stability in specific applications like oral delivery [132]. Finally, PLGA is best at a narrow range of functional indicators and is most promising in sustained-release formulations [134]. This analysis explains the popularity of LNPs as the standard in RNA vaccines while highlighting the niche benefits of alternative delivery platforms [140].

### 6.1. Engineering Constraints of RNA Nanocarriers

The clinical translation of nanocarriers is highly impacted by engineering constraints, with chemical stability being a primary factor [132,140]. LNPs and NLCs are structurally robust, and their stability is highly dependent on their physical state; for instance, lyophilized NLCs have demonstrated long-term stability at room temperature for over 8 months [132]. In contrast, poly(lactic-co-glycolic acid) (PLGA) undergoes hydrolytic degradation (hydrolysis) in aqueous environments, where the cleavage of ester linkages can create an acidic microenvironment that potentially degrades the encapsulated cargo [129,138]. Exosomes are particularly sensitive and typically require storage at −80 °C to prevent membrane rupture, cargo leakage, or loss of biological activity [131,141].

Cold-chain requirements further classify these platforms. LNPs generally require ultra-cold storage for long-term stability, with the BNT162b2 vaccine requiring −60 °C to −80 °C, while the mRNA-1273 vaccine is stored between −15 °C and −25 °C [25,142]. SLNs and NLCs offer significantly more flexibility, often remaining stable at temperatures between 4 °C and 25 °C, especially when converted into solid powders [132,133]. Exosomes require consistent long-term storage at −80 °C to maintain integrity, while PLGA formulations are often stored long-term at −20 °C to mitigate the onset of hydrolysis [131,143].

A critical differentiator is compatibility with lyophilization (freeze-drying). LNPs, SLNs, and NLCs are highly compatible with freeze-drying when utilizing appropriate cryoprotectants, such as sucrose or trehalose, to maintain nanoparticle size and integrity [132,143]. Similarly, PLGA particles are standardly dried via lyophilization during their final formulation stage to ensure long-term stability [129]. In contrast, exosomes remain challenging to lyophilize, as the process often induces aggregation and significantly reduces encapsulation efficiency [131]. The physicochemical stability of LNPs and NLCs can be verified for 6–12 months, whereas PLGA typically maintains integrity for 3–6 months, and non-engineered exosomes are most restricted, often limited to 1–3 months of viable shelf life [132,143]. This establishes SLNs/NLCs as the most flexible for global logistics, while exosomes remain the most challenging regarding the cold chain.

The stability analysis confirms the effectiveness of LNPs and SLNs/NLCs over PLGA and exosomes for real-life applications, particularly global vaccine distribution. The ability to achieve thermostability through lyophilization represents a significant opportunity for LNPs and NLCs [132,143], while the stringent storage requirements of exosomes remain a substantial hurdle for their clinical scaling, despite their inherent biological advantages [131].

### 6.2. Translational and Manufacturing Considerations

There is a significant contrast between bench-scale development and the real-life clinical application of nanocarriers. LNPs demonstrate superior large-scale production capabilities through the use of microfluidic technology, which ensures high reproducibility and scalability by precisely controlling the mixing of lipid and aqueous phases [25,139,140]. In comparison, SLNs and NLCs are typically manufactured using the hot high-pressure homogenization (HPH) method, a powerful and reliable technique for commercial-scale production that is easy to scale up and avoids toxic solvents [132,133,144].PLGA particles are standardly produced via the emulsion-solvent evaporation method, which is well established for industrial scaling, although it is technically more complex than LNP microfluidics [129,138]. On the other hand, exosomes face inherent limitations regarding scale-up due to the complex and time-consuming cell culture methods and the lack of standardized high-throughput isolation protocols required for their production [131,141,145].

The platforms vary significantly regarding reproducibility and good manufacturing practice (GMP) compliance [140,141]. LNPs and PLGA benefit from established manufacturing processes and clear regulatory pathways [134,136]. While SLNs and NLCs are mature in the cosmetics industry (with ~40 products already marketed), they require further validation for RNA-specific applications to meet stringent pharmaceutical standards [133,144]. Exosomes present the greatest challenge, as they exhibit significant batch-to-batch variability and lack standardized protocols for reliable characterization and isolation [124,137].

From a cost perspective, exosomes are the most expensive delivery platform because of the technical complexities involved in their extraction and purification [131,137]. LNPs also incur high costs due to the specialized ionizable lipids required for their formulation [140,142]. PLGA is generally more expensive than lipid-based carriers such as SLNs/NLCs due to the cost of polymer synthesis [146]. Consequently, SLNs and NLCs are the most affordable of the four platforms in terms of raw materials and production costs [146]. The maturity of regulations completes the translational picture: LNPs are FDA-approved for the delivery of both siRNA drugs (Onpattro) and mRNA vaccines [25,136]. PLGA has long-standing approval for various drug delivery products [138], whereas SLN/NLC-based products have primarily reached the market for topical or dermal applications [133]. Currently, exosomes have no clinical approvals, as they remain in the early stages of clinical evaluation [124,131]. Table 4 compares and summarizes the multiple parameters of LNPs, SLNs, NLCs, PLGA, and natural (exosome) nanocarriers.

**Figure 5:**
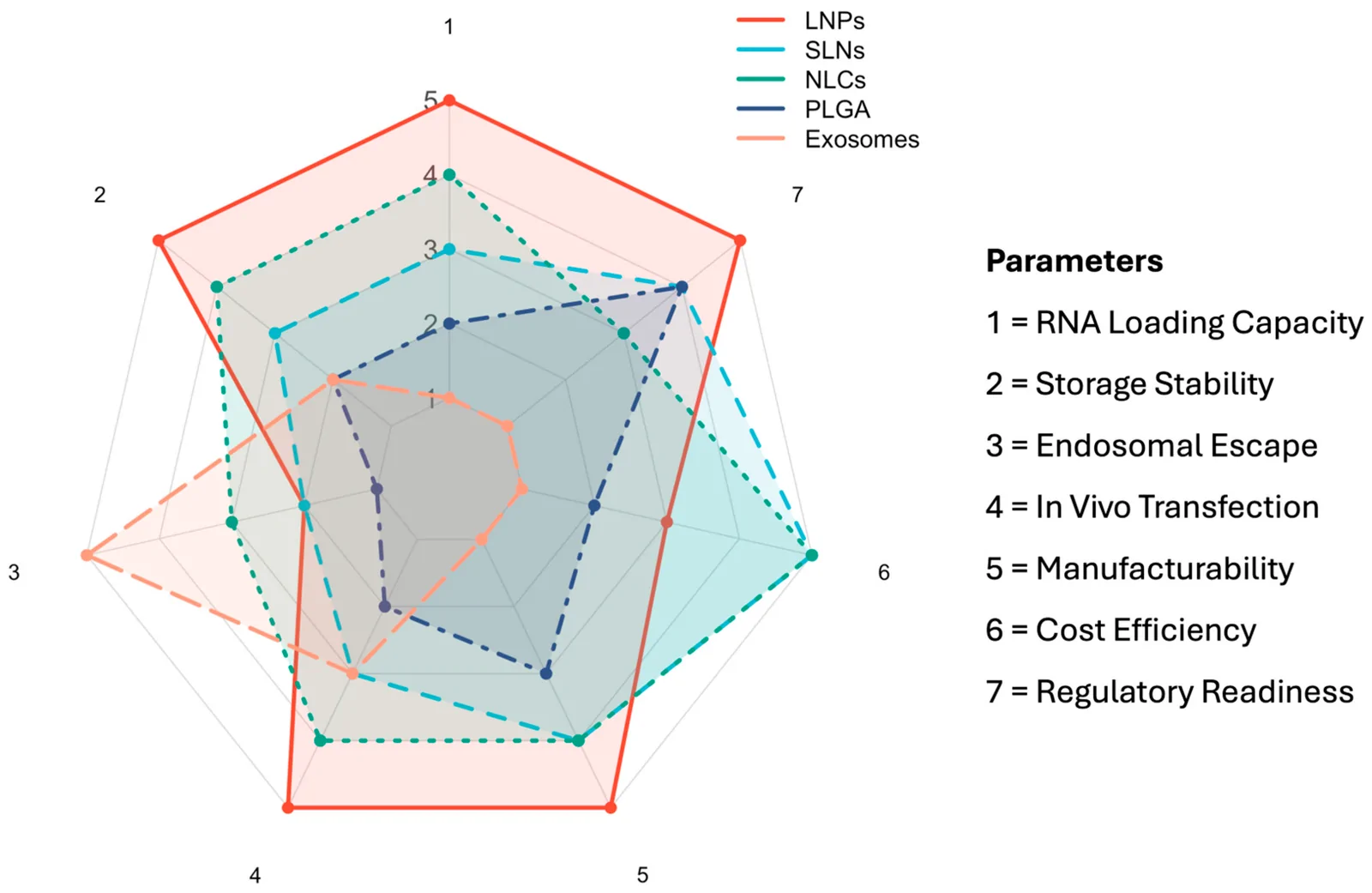
Displays a radar chart comparing seven different parameters, including RNA loading capacity, storage stability, endosomal escape, in vivo transfection, manufacturing, cost efficiency, and regulatory readiness for LNPs, SLNs, NLCs, PLGA, and exosome nanocarriers. This radar plot is a qualitative indication from published literature translated into a standardized scoring system to facilitate visual comparison. Scores reflect relative performance, not absolute or directly comparable measurements across studies. Each parameter was assigned a score from 1 to 5 using the following qualitative scale: 1 = very low/poor performance, 2 = limited performance, 3 = moderate or variable performance, 4 = good performance, 5 = high or consistently strong performance.

The translational analysis indicates a clear hierarchy. LNPs are the most clinically developed with fully established regulatory paths, explaining their central role in the success of RNA vaccines [25]. PLGA remains a robust, well-profiled platform but is not currently optimized for rapid response RNA vaccine needs [129]. SLNs/NLCs and exosomes are promising but are currently restricted by limitations in production, standardization, and regulatory maturity [124,133]. Figure 5 shows a radar plot comparing seven different parameters of the five nanocarriers.

## 7. Manufacturing and Scale-Up Challenges of Nanocarrier Platforms for RNA Vaccines

The global rollout of mRNA vaccines, delivered worldwide to billions of people, has firmly established LNPs as a practical and scalable delivery vehicle for nucleic acid therapeutics. At its core, what made this possible was the compatibility of cell-free mRNA synthesis with ionizable lipid carriers that simultaneously shield polyanionic RNA from enzymatic degradation and trigger endosomal release within target cells [147]. However, the infrastructure built to meet a single, urgent pandemic need is not straightforwardly transferable to the next generation of mRNA-based medicines. Moving into personalized cancer immunotherapy and treatments for rare diseases requires a degree of manufacturing flexibility and precision that pandemic-era platforms were never designed to provide [148]. Three interrelated obstacles stand out in this context: navigating increasingly exacting regulatory frameworks, achieving reliable batch-to-batch reproducibility, and reconciling the practical trade-offs between precision microfluidic systems and high-throughput turbulent mixing approaches [149].

### 7.1. GMP Considerations and Regulatory Expectations

Producing mRNA-LNP drug products at GMP standard is substantially more demanding than working at the bench, not merely because of scale, but because every chemical, manufacturing, and control (CMC) parameter must be rigorously defined and controlled [147,150]. Both the U.S. FDA and the EMA now expect manufacturers to prospectively identify and define their critical quality attributes (CQAs), covering everything from lipid identity and purity through to encapsulation efficiency and RNA structural integrity before clinical batches are released [148,151]. One of the more technically challenging requirements is demonstrating that a formulation remains comparable after scale-up: even relatively minor changes in mixer geometry or the nitrogen-to-phosphate (N/P) ratio can compromise the reliability of predictions made from small-scale models [149,152]. The challenge becomes still more acute for personalized cancer vaccines, where the model is not scale-up but rather “scale-out,” running dozens of end-to-end parallel manufacturing lines simultaneously, each for a different patient-specific antigen sequence. Such a model cannot rely on traditional batch-size comparability studies; it requires entirely new frameworks for potency assessment and real-time release testing [147,152].

**Figure 6:**
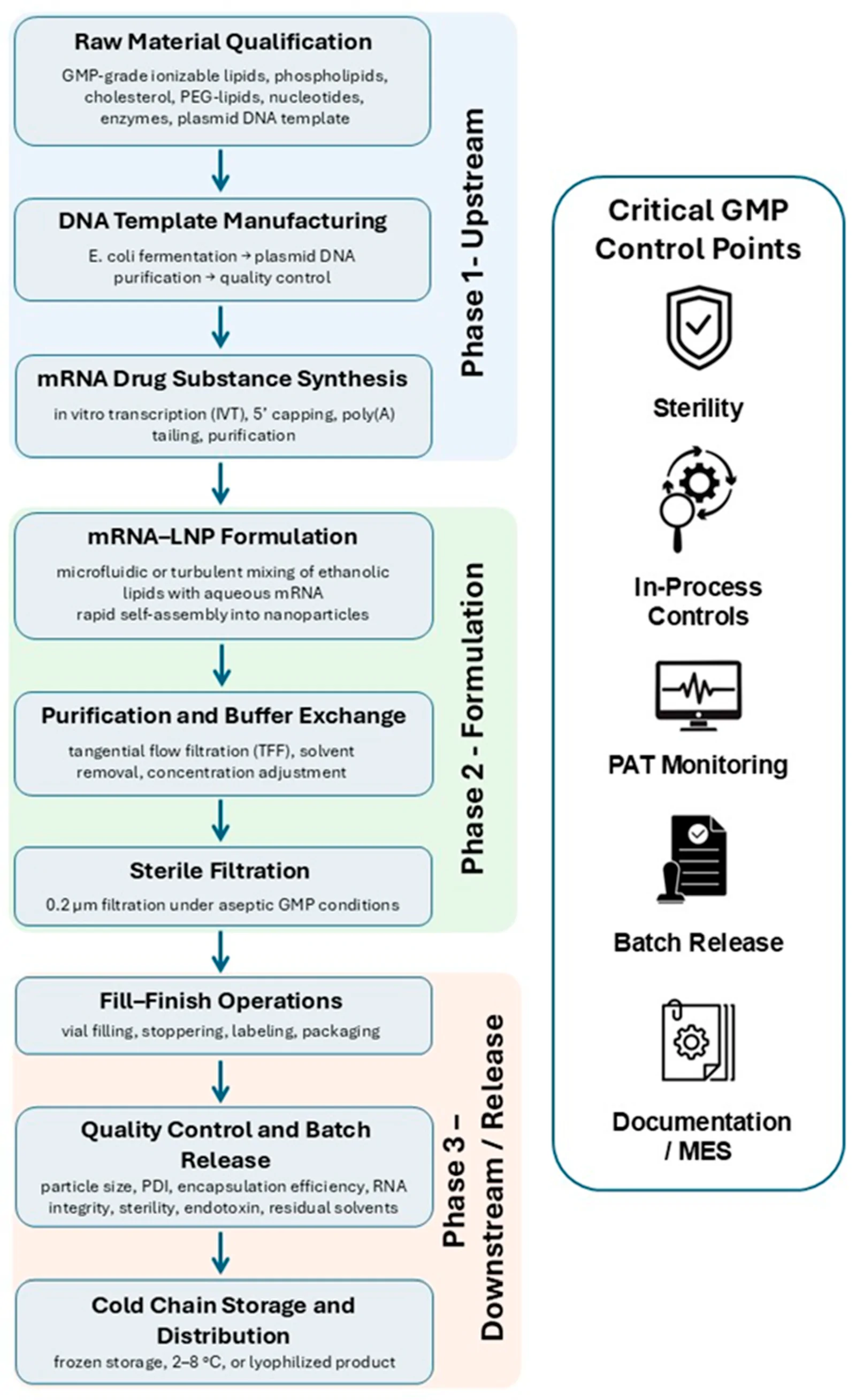
GMP manufacturing workflow for mRNA-LNP vaccines. Schematic flowchart illustrating the end-to-end GMP manufacturing process, organized into upstream, formulation, and downstream/release phases. The workflow spans raw material qualification, plasmid DNA template production, in vitro transcription, nanoparticle formulation, purification, sterility, in-process controls, PAT monitoring, batch release, and documentation systems.

Regulatory agencies have, to some extent, tried to keep pace with this complexity through “platform-based” review frameworks, wherein accumulated experience with an established mRNA-LNP system can be leveraged to expedite assessment of new therapeutic targets built on the same architecture [147]. Even within these streamlined pathways, however, applicants remain obligated to provide product-specific characterization of their starting materials, particularly the linear DNA template, which must be shown to have an intact coding sequence and a correctly defined poly(A) tail region. A schematic overview of the end-to-end GMP manufacturing workflow for mRNA–LNP vaccines, including the major process phases and critical control checkpoints, is presented in Figure 6.

### 7.2. Batch Reproducibility and Process Robustness

LNP formation depends on nucleation-limited self-assembly, a process that unfolds over milliseconds as ethanolic lipid solutions meet aqueous RNA streams, making it exquisitely sensitive to any perturbation in the mixing environment [151,152]. In practice, controlling variability means optimizing critical process parameters (CPPs), including the flow rate ratio (FRR), total flow rate (TFR), and the precise composition of the solvent system [149,153]. When these parameters are properly secured through Design-of-Experiment (DoE) approaches, it is possible to maintain particle diameters consistently within a defined target window (for example, 60–180 nm) with minimal variation across batches that differ substantially in overall scale [151,154]. Some studies have also incorporated low-frequency sonication into continuous-flow production systems as a means of both monitoring particle characteristics in real time and ensuring that only LNPs within the desired size range are harvested, which is a practical approach to maintaining process robustness and product quality across the bench-to-manufacturing transition [154]. At commercial volumes, maintaining this level of process control requires more than careful parameter optimization; it demands real-time monitoring infrastructure. Process Analytical Technologies (PAT) have become central to this effort [60]. In-line dynamic light scattering (DLS) probes allow continuous tracking of particle size during a run, while UV-vis spectrophotometry provides a real-time window on encapsulation efficiency without interrupting the process, enabling immediate feedback control [155]. These feedback systems are not merely convenient at manufacturing scale; by identifying and correcting deviations within minutes of startup, they can meaningfully reduce material waste associated with out-of-specification production [155].

### 7.3. Quality Control and Product Characterization

What makes LNP quality control particularly demanding is that subtle changes in mixing kinetics can affect clinically relevant properties, including biodistribution, cellular uptake, and endosomal escape, which are not always predictable from straightforward physical measurements [149]. PDI is the standard starting point for characterization, with pharmaceutical-grade preparations generally targeting PDI values below roughly 0.2–0.3 as an indicator of population homogeneity [154]. Encapsulation efficiency is equally important: well-optimized formulations often exceed ~85% entrapment, helping to reduce the proportion of free RNA vulnerable to nuclease-mediated degradation during storage and in vivo studies [152,154].

Physical characterization alone is, however, insufficient. RNA purity and integrity must also be verified after formulation, since suboptimal encapsulation conditions can contribute to RNA degradation. Of particular concern are dsRNA contaminants and truncated mRNA species carried over from the in vitro transcription (IVT) step: both can activate innate immune sensors and, in the case of truncated fragments, compete with full-length mRNA for lipid-binding sites, reducing effective delivery [156]. Beyond this, confirming lipid composition and measuring the apparent pKa of the ionizable lipid component are necessary to predict how the LNP will behave at physiological and endosomal pH, both of which influence in vivo stability and transfection activity [151]. Finally, residual ethanol from the formulation process must be reduced to pharmacopeially acceptable levels through tangential flow filtration (TFF) or dialysis; failure to do so introduces safety concerns for parenteral administration [154].

### 7.4. Microfluidics Versus Bulk Manufacturing: Scalability Challenges

The two dominant mixing strategies used in LNP production today, microfluidic-based “scale-out” and turbulent flow “scale-up,” approach the reproducibility–throughput trade-off from opposite directions, but neither fully eliminates it [86]. Microfluidic devices, whether they employ staggered herringbone (SHM) or toroidal channel geometries, are widely regarded as a standard for formulation precision. These systems produce highly monodisperse populations through millisecond-scale, nanoliter-volume mixing events that reduce stochastic variability [86,152]. The main limitation is throughput; a single microfluidic channel is typically constrained to roughly 0.1 L/h [86,152]. Parallelization is one practical solution, and the SCALAR (Silicon sCAlable Lipid nAnoparticle geneRation) platform illustrates how far this approach can be pushed: by integrating 256 discrete mixing units on a single chip, the system achieves 17 L/h throughput while maintaining the particle characteristics established at the screening scale [86,152]. Turbulent mixing systems, including T-junctions, confined impinging jet (CIJ) mixers, and multi-inlet vortex mixers (MIVM), work on an entirely different principle, exploiting high-Reynolds-number flow to generate rapid supersaturation and particle nucleation within approximately 1.5 ms [157]. These devices can sustain production of uniform LNPs at rates up to 5 L/min, making them a practical choice for pandemic-scale or other high-volume applications [86,151,157]. Their significant limitation is the inverse of microfluidics: scaling down for early-discovery work, where only milligram quantities are needed, is technically difficult because the turbulent regime required for efficient mixing may not be maintained at very low flow rates [86,157].

Even microfluidic systems face engineering challenges beyond throughput. Surface fouling, caused by the gradual adsorption of lipid components onto channel walls, can lead to progressive clogging, rising PDI, and eventual process failure, often within minutes of starting a run [151,158]. A promising solution has emerged in the form of immobilized liquid lubricant coatings: applying perfluorodecalin to channel surfaces creates an antifouling interface that, in documented cases, extends stable operating time from a few minutes to well over 180 min [158]. Separately, crossflow micromixing using stainless-steel membrane architectures has demonstrated scalability to 500 mL/min while preserving the CQAs established at smaller scales, providing a potentially important bridge between the precision of microfluidics and the throughput demands of manufacturing [153]. Taken together, these developments reinforce a common theme: there is no single universal solution for RNA nanocarrier scale-up, and success depends on matching the manufacturing approach to the specific clinical application, accepting different trade-offs accordingly.

## 8. Current Challenges

### 8.1. Intrinsic Instability of RNA and Sensitivity to Formulation and Handling Conditions

RNA is a fragile macromolecule. Its phosphodiester backbone and nucleobases are vulnerable to hydrolytic cleavage, oxidative modification, and enzymatic degradation by nucleases, and the kinetics of these reactions depend heavily on how the material is handled and on the physicochemical environment. Agitation, freeze–thaw cycling, light exposure, pH, buffer composition, ionic strength, residual trace metals, and dissolved oxygen each accelerate degradation to varying degrees, and in practice, the contribution of each factor is rarely separable at the formulation stage.

In nanocarrier formulations, RNA stability is tightly coupled to the chemical and structural integrity of the delivery vehicle itself. Lipid oxidation and hydrolysis have been documented across a growing body of recent work [92,94,144], and these reactions rarely occur in isolation. Payload leakage, depletion of PEG-lipid from the particle surface, particle fusion and aggregation, and lipid hydrolysis are interrelated events that together compromise the encapsulated RNA. The practical consequence is greater water ingress into the particle core, which, in turn, drives strand breakage and damage to the 5′ cap and poly(A) tail, two features that are indispensable for translational competence.

Crucially, the loss of potency that follows does not always track with conventional bulk-formulation integrity assays. Reactive contaminants generated from lipid excipients can chemically modify the encapsulated RNA even in formulations that, by standard analytical criteria, appear physically intact at release. On that basis, Kang and colleagues [159] argue that lipid quality itself should be treated as a primary critical-stability attribute rather than a secondary material concern.

### 8.2. Standardizing Analytical Methods ACROSS Platforms

Efforts to standardize assessment are constrained, first, by the sheer number and heterogeneity of critical quality attributes (CQAs) that must be characterized for both the RNA cargo and its nanocarrier. On the RNA side, sponsors are expected to document sequence identity, strand-length distribution and truncation, variability of the 5′ cap and poly(A) tail, double-stranded RNA content, and a range of process-related impurities. On the carrier side, particle size distribution, morphology, encapsulation efficiency, and lipid degradation products all factor into the profile [160,161,162].

Sample preparation introduces a further layer of complication that is often underestimated on the bench. Choices around detergents, organic solvents, and denaturation conditions prior to analysis can systematically bias both the RNA readout and the measured nanostructure of the formulation. Beyond this, the orthogonal platforms routinely used for characterization capillary gel and capillary electrophoresis (CGE/CE), several modes of liquid chromatography (LC), LC–mass spectrometry (LC–MS), next-generation sequencing, dynamic light scattering and nanoparticle tracking analysis (DLS/NTA), and cryogenic electron microscopy (cryo-EM) differ substantially in sensitivity, throughput, and suitability for routine quality control [162,163].

Regulatory frameworks addressing this space continue to emerge, notably from the World Health Organization, the EMA and the European Directorate for the Quality of Medicines & HealthCare. Even so, the absence of widely accepted reference standards and the limited evidence for inter-laboratory comparability remain fundamental obstacles to uniform quality assessment across the sector.

### 8.3. Bridging the Gap Between Analytical Stability and Functional Potency

Real-time potency and immunogenicity do not always track with analytical stability. Concurrent degradation pathways can produce measurable declines in biological efficacy well before any noticeable shift in bulk physicochemical parameters, mean particle size, encapsulation efficiency, or total lipid content is registered. Both Kang et al. [164] and Nomani et al. [92] report that expression is substantially reduced by the formation of RNA-lipid adducts and by the accumulation of lipid oxidation products, even when bulk particle metrics and the electrophoretically determined full-length RNA fraction remain largely unchanged. Small changes in the particle’s internal nanostructure, often invisible to routine analytics, can also alter endosomal escape and subsequent translational efficiency.

A more predictive analytical paradigm is therefore increasingly favored in practice: one that pairs orthogonal physicochemical characterization with cell-based functional potency assays, targeted forced-degradation studies, and stress profiling [160,161]. The aim of these multimodal approaches is to map the molecular hierarchy of failure routes and to inform therapeutically meaningful acceptance criteria for release and stability testing of RNA–LNP products.

### 8.4. Evolving Regulatory Frameworks for Diverse RNA Modalities

Regulatory expectations in this area are tightening, but both agencies and sponsors still draw most of their operational experience from the early development of mRNA–LNP products. saRNA, circRNA, and a growing family of non-lipid nanocarrier systems are now entering the therapeutic pipeline, and several key regulatory questions remain unresolved, among them the choice of suitable potency frameworks, the boundaries of permissible comparability, and the type and volume of data needed to support manufacturing changes, thermostable reformulations, and annual variant or antigen updates [147]. Platform-oriented lifecycle tools, such as those outlined in ICH Q12, are increasingly being used to structure these decisions.

Regulatory mechanisms intended to ease follow-on development have emerged on both sides of the Atlantic: the master-file concepts under development in the EU, and the Platform Technology Designation program recently articulated by the U.S. FDA, both point toward a more platform-centric approach to approval. Bridging these mechanisms in practice will require highly sensitive analytics paired with a disciplined, risk-based framework consistent with the most recent agency guidance [35].

## 9. Future Direction

**Figure 7:**
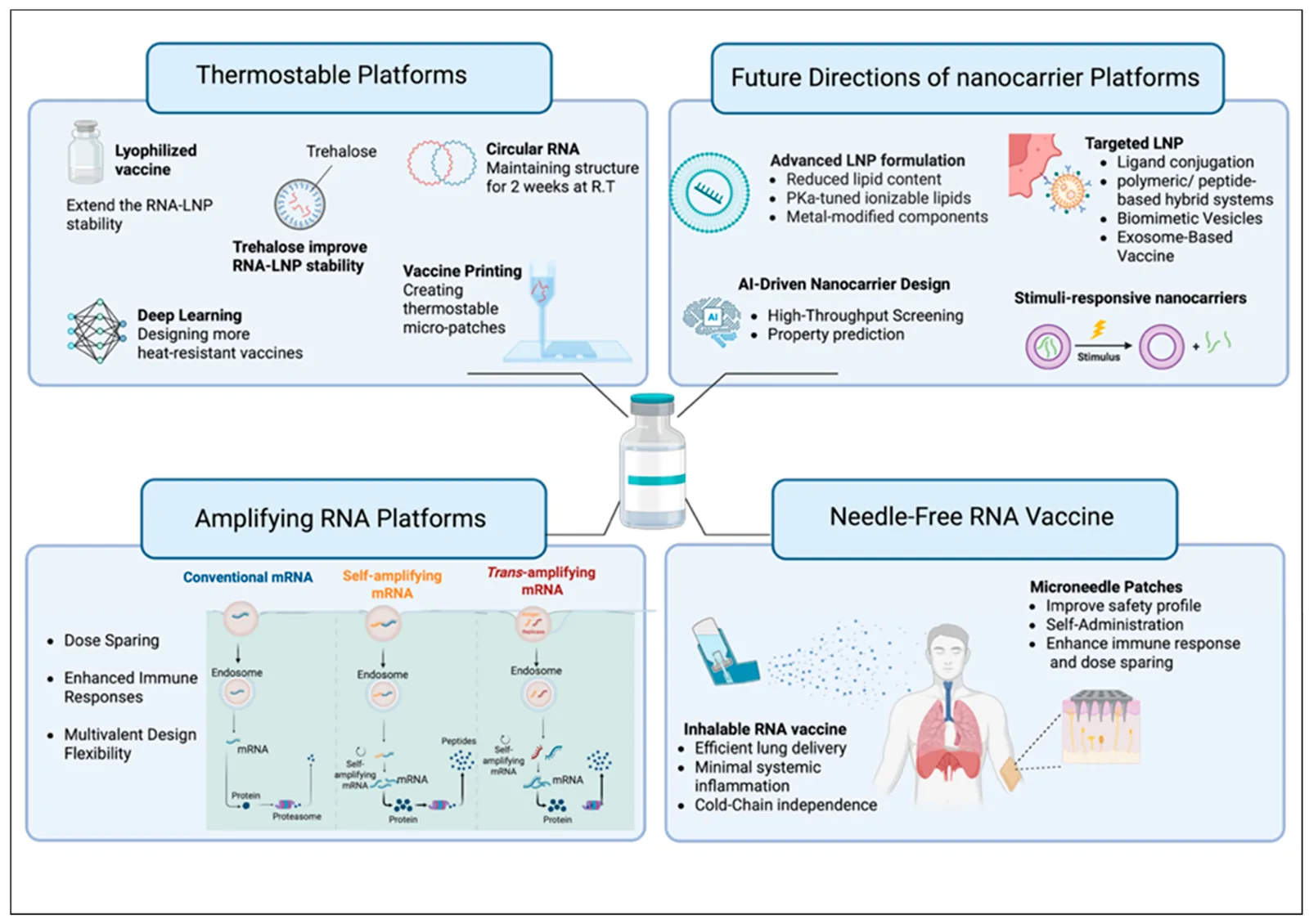
Emerging platforms and technologies advancing RNA vaccine development. This schematic provides an overview of key advancements in RNA vaccine development, addressing limitations in stability, dosing, delivery, and nanocarrier optimization. (**Top Left**) Thermostable Platforms: Strategies to overcome cold-chain reliance include the use of circular RNA, lyophilized formulations, and trehalose-enhanced lipid nanoparticles. Additionally, computational guidance via deep-learning degradation models further facilitates the design of heat-resistant sequences. (**Bottom Left**) Self-amplifying RNA Platforms: Advancements in saRNA and trans-amplifying RNA (taRNA) architectures enable significant dose-sparing and low immunogenicity through the reduction in antigen dosages. Multivalent designs expand strain coverage for infectious diseases. (**Top Right**) Future Nanocarrier Directions: These advanced delivery systems offer improved targeting and safety. Innovations include pKa-tuned ionizable lipids for better endosomal escape, biomimetic and exosome-based carriers for enhanced biocompatibility and tissue targeting, and stimuli-responsive nanocarriers that release their payload in response to disease-specific microenvironments. (**Bottom Right**) Mucosal and Needle-Free Delivery: Transitioning from intramuscular injections, these platforms utilize inhalable LNP powders for pulmonary delivery and automated microneedle vaccine-printing systems. These patches provide a decentralized, thermostable option that preserves mRNA functionality and elicits durable immune responses. Alharthi, S. (2026) https://BioRender.com/n1rq2cb, accessed on 7 July 2026.

The future of RNA vaccines is shaped by rapid advances in formulation science, delivery platforms, and computational design. Emerging technologies aim to overcome current limitations related to stability, targeting, and scalability by integrating smart nanocarriers, alternative RNA architectures, and data-driven optimization strategies, as shown in Figure 7. These developments are expected to expand the clinical applicability of RNA vaccines beyond infectious diseases, enabling more precise, durable, and accessible interventions across oncology, personalized medicine, and global health.

### 9.1. Thermostable RNA Vaccine

Vaccine researchers have been working on improving the thermal stability of RNA platforms to help reduce cold-chain constraints in recent years [165,166,167,168]. In this field, circRNA platforms have emerged, as their circular structure allows resistance to degradation, reinforcing functional efficacy and protein production, even when stored at room temperature for more than two weeks [167]. Similarly, modifying the design of LNPs can improve the durability of linear mRNA vaccines, allowing them to be stored at 25 °C for a week, resulting in promoting flexible distribution [168]. Although freeze-dried mRNA-LNP vaccines can maintain their physical and chemical stability and biological activity for several months at room temperature, this approach faces challenges due to a lack of long-term safety data on humans, technical complexity, and the high cost of freeze-drying on an industrial scale [165]. In addition to the developments at the molecular level, microneedle vaccines have become a viable delivery method that could help decrease the need for cold-chain storage [167]. Trehalose-enhanced LNPs further improve mRNA stability and help narrow the gap between in vitro and in vivo performance [169]. Ultimately, deep-learning models for RNA degradation provide valuable guidance in designing heat-resistant vaccines, but they still require further structural refinement to improve biological accuracy [169].

### 9.2. Amplifying RNA Platforms

After studies in mice showed that a single dose of saRNA-LNP can stimulate a strong antibody response, the saRNA is receiving a lot of attention. These findings also suggest that even lower doses can still trigger a strong immune response [170]. Further studies confirmed these findings, showing that the vaccine was safe and provided strong protection against multiple variants in mice, rats, and hamsters [171]. Additional confirmation was obtained in non-human primates, where low doses significantly reduced viral replication throughout the respiratory tract [172]. Also, influenza studies showed that using a single saRNA construct to express two antigens broadened strain coverage and improved protection in ferrets [173]. Studies of the ARCT-154 vaccine showed strong immune responses and protection against COVID-19, while separate work found that modified saRNA was more stable and produced more consistent responses in vivo [174,175]. In contrast to monolithic saRNA vectors, the taRNA platform employs a two-component strategy comprising a replicase-encoding non-replicating nrRNA and an antigen-encoding transreplicon (Figure 7). Recent preclinical studies of taRNA-based SARS-CoV-2 vaccines showed that they produced antibody levels similar to those of standard mRNA vaccines, while using 40 times less antigen.

### 9.3. Mucosal and Needle-Free RNA Vaccine Delivery

RNA vaccine delivery strategies are increasingly shifting toward mucosal and needle-free platforms to improve patient compliance and accessibility. Inhalable LNP formulations have demonstrated efficient pulmonary mRNA delivery with minimal inflammation in preclinical models [176]. Spray-freeze-drying approaches incorporating cryoprotectants have enabled the generation of stable, inhalable mRNA–LNP dry powders with in vivo delivery efficiency comparable to liquid formulations [177].

Microneedle-based transdermal delivery systems represent an additional needle-free alternative (Figure 7). Automated microneedle vaccine printers have been used to fabricate thermostable mRNA-loaded microneedle patches (MNPs) that preserve LNP structure and mRNA functionality following drying. In murine models, these patches elicited durable humoral and cellular immune responses comparable to intramuscular injection when encoding the SARS-CoV-2 spike RBD, with shelf stability of at least 6 months at room temperature [167]. Clinical feasibility has been demonstrated in a Phase I trial (NCT05315362), in which nanoporous microneedle patches safely delivered fractional intradermal doses of mRNA-1273 or BNT162b2 vaccines. Although antibody responses were limited due to low mRNA loading, the study confirmed safety and highlighted optimization opportunities for future development [178].

### 9.4. Future Directions of Nanocarrier Platforms

Future RNA vaccine development will rely on continued optimization of nanocarrier systems to improve delivery efficiency, tissue targeting, and safety (Figure 7). Advanced LNP formulations incorporating reduced lipid content, pKa-tuned ionizable lipids, or metal-modified components have shown enhanced endosomal escape and reduced inflammatory responses [179]. Targeted delivery strategies increasingly employ ligand conjugation, polymeric or peptide-based hybrid systems, and biomimetic vesicles that mimic extracellular vesicles to enhance tissue specificity and immune evasion. Exosome-based carriers further offer natural tropism and improved biocompatibility [180]. Clinical translation of advanced nanocarriers is exemplified by the Phase I BNT211-01 trial, in which CLDN6-specific CAR-T cells were combined with an amplifying RNA vaccine (CARVac). This approach demonstrated acceptable safety, prolonged CAR-T cell persistence, and preliminary antitumor activity in patients with CLDN6-positive solid tumors [180].

Stimuli-responsive nanocarriers represent an emerging area of interest, enabling controlled RNA release in response to disease-specific microenvironmental cues such as acidic pH or redox conditions [181]. Preclinical tumor models have demonstrated preferential accumulation and triggered the release of p53-encoding mRNA, resulting in restored protein expression [182]. However, most clinically advanced RNA vaccines continue to rely on conventional or optimized LNPs, with fully stimuli-responsive systems largely remaining at the preclinical stage [181].

Artificial intelligence and machine learning are increasingly applied to nanocarrier design by enabling high-throughput screening of lipid libraries and the prediction of physicochemical and biological properties. AI-optimized ionizable lipids have demonstrated delivery efficiencies comparable to or superior to established benchmarks such as MC3 and SM-102 [183]. This data-driven design paradigm, already advancing in gene therapy applications such as NTLA-2001, is expected to play a key role in the development of next-generation RNA vaccines [184].

## Figures and Tables

**Table 1:** Comparative summary of major nanocarrier platforms used for RNA delivery. The table summarizes their structural composition, advantages, limitations, endosomal escape mechanisms, and current stage of clinical development. Differences in physicochemical architecture affect RNA loading capacity intracellularly and its translational applicability. Adapted from [17,18,30,58].

Platform	Structural Composition & Release Mechanism	Key Advantages & Transfection Efficiency	Major Limitations & Storage Stability	Endosomal Escape Mechanism	Clinical Maturity & Representative products
Lipid Nanoparticles (LNPs)	Ionizable lipid + helper phospholipid + cholesterol + PEG-lipid. Release via pH-triggered disassembly.	High encapsulation efficiency, excellent transfection across diverse cell types, clinically validated.	Cold-chain dependence, anti-PEG antibodies, and liver tropism bias. Poor thermal stability (requires −20 °C to −80 °C).	pH-dependent ionization, membrane destabilization, and lipid mixing.	Clinically approved. Comirnaty^®^ (Pfizer (New York, NY, USA)/BioNTech (Mainz, Germany)), Spikevax^®^ (Moderna (Cambridge, MA, USA)).
Solid Lipid Nanoparticles (SLNs)	Solid crystalline lipid core stabilized by surfactants. Release via slow erosion and passive diffusion.	Improved physical stability, reduced lipid mobility, and established excipients. Moderate transfection efficiency.	Limited RNA loading, polymorphic transitions, and possible payload expulsion. Good ambient/refrigerated storage stability.	Limited intrinsic escape often requires cationic additives.	Preclinical/early translational. No approved RNA products to date.
Nanostructured Lipid Carriers (NLCs)	A mixture of solid + liquid lipids forming a disordered matrix. Release via uniform diffusion from fluid pockets.	Increased free volume, improved loading capacity, and enhanced thermostability. Moderate to high transfection efficiency.	Formulation complexity and regulatory standardization challenges. High storage stability resists polymorphic transitions.	Improved release due to matrix disorder and lipid mixing.	Preclinical/emerging. In ongoing clinical vaccine candidates.
Polymer-Based Nanocarriers (Polyplexes)	Cationic/ionizable polymers (e.g., PEI, chitosan, CARTs, Janus dendrimers). Release via charge alteration or matrix degradation.	High tunability, controlled degradation, and adjustable charge density. High to moderate transfection in vitro, variable in vivo.	Cytotoxicity risk, immune activation, and batch variability. The assembly–disassembly paradox. Generally stable at 4 °C (dependent on polymer type).	Proton sponge effect.	Mostly preclinical. Exonys^®^ (Gyeonggi-Do, Republic of Korea) polymers used in active clinical gene delivery trials.
Hybrid Nanocarriers	Polymeric core + lipid shell. Release via core degradation following shell fusion.	Combined structural stability and biocompatibility, enhanced protection. High transfection efficiency.	Manufacturing complexity and scalability issues. Moderate storage stability (varies by core/shell components).	Combined proton sponge + membrane fusion.	Preclinical. Experimental oncology pipelines.
Exosome-Like/Biomimetic Systems	Natural or engineered extracellular vesicles. Release via native membrane fusion.	Immune evasion, inherent targeting, and biological compatibility. Donor-dependent transfection efficiency.	Difficult large-scale production, loading inefficiency, and regulatory ambiguity. Requires ultra-low storage temperatures.	Natural membrane fusion pathways.	Early research. ExoIL-12 (clinical trials).

**Table 2:** RNA-lipid nanoparticle formulation components, functional roles, and immunocompatibility.

Component Class	Representative Examples	Functional Role in RNA-LNPs	Relevance to Performance and Immunocompatibility	Reference
Ionizable lipid	ALC-0315, SM-102, DLin-MC3-DMA	Drives RNA complexation during assembly and promotes endosomal escape after uptake	Near-neutrality at physiological pH limits nonspecific protein adsorption, complement activation, and systemic toxicity, while protonation in endosomes enables membrane destabilization and cytosolic RNA release.	[64,65,66].
Helper phospholipid	DSPC, DOPE	Regulates membrane organization, interfacial stability, and fusogenicity	Influences nanoparticle structure, intracellular trafficking, and delivery efficiency; phospholipid identity can shift the balance between structural stability and membrane fusion.	[40,67,68].
Sterol	Cholesterol, cholesterol analogs	Enhances membrane packing, rigidity, and phase behavior	Supports particle integrity and membrane fusion, while sterol identity and content can alter biodistribution, tropism, and transfection performance.	[40,67,68].
PEG-lipid	ALC-0159, DMG-PEG2000, DSPE-PEG2000	Controls particle size and provides steric surface stabilization	Reduces aggregation, protein corona formation, and mononuclear phagocyte system recognition; PEG structure and anchor chemistry also affect uptake and expression.	[67,69,70,71].
Modified RNA cargo	N1-methyl-pseudouridine (m1Ψ)-modified mRNA	Improves RNA stability and translational efficiency	Reduces recognition by innate immune sensors, including TLR3, TLR7/8, RIG-I, and MDA5, thereby suppressing inflammatory signaling and improving protein expression.	[72,73,74].
Acidic assembly buffer	Citrate, acetate	Enables rapid self-assembly during microfluidic mixing by protonating ionizable lipids	Critical for efficient RNA encapsulation and particle formation; buffer conditions can influence particle morphology and downstream biological activity.	[75,76,77].
Final formulation buffer	Tris, phosphate, histidine	Maintains near-neutral pH and formulation stability after assembly	Preserves colloidal integrity, limits hydrolysis and oxidation, and improves storage stability; buffer composition can also affect uptake and transfection.	[78,79,80].
Cryoprotectant/stabilizer	Sucrose, Trehalose	Protects nanoparticles during freezing, thawing, and storage	Improves stress tolerance and shelf stability by reducing aggregation and structural disruption.	[79]
Membrane-active additive	Esterified fatty acids	Further stabilizes membrane structure and reduces leakage	May improve nanoparticle stability and immunocompatibility by tightening membrane packing.	[67]

**Table 3:** Stability engineering strategies for RNA vaccine nanocarriers, linking degradation mechanisms to targeted interventions and their impact on RNA and carrier stability.

Stability Challenge	Degradation Mechanism	Engineering Strategy	Impact on Stability	Reference
RNA hydrolysis	Phosphodiester cleavage	Nucleoside modification, sequence optimization	Prolonged RNA half-life	[92]
Oxidative damage	Lipid/RNA oxidation	Antioxidants, oxygen-controlled processing	Reduced chemical degradation	[93,94]
Aggregation	Particle fusion	Lipid ratio optimization	Maintained particle size	[92,93]
Freeze–thaw stress	Ice crystal damage	Cryoprotectants (trehalose, sucrose)	Improved physical stability	[95]
Lyophilization stress	Dehydration-induced collapse	Optimized freeze-dry cycles, bulking agents	Enhanced storage stability	[94,96,97,98]
Thermal instability	Lipid phase transitions	NLCs, ionizable lipid redesign	Improved thermostability	[99]
Enzymatic degradation	RNase exposure	Encapsulation efficiency, sterile processing	Preserved RNA integrity	[54,93]

**Table 4:** Comparative matrix of functional performance, stability, and manufacturing parameters for synthetic and natural nanocarriers. This table provides a cross-platform evaluation of lipid-based (LNPs, SLNs, NLCs), polymeric (PLGA), and natural (exosome) nanocarriers.

Parameter	LNPs	SLNs	NLCs	PLGA Nanoparticles	Exosomes	Reference
Encapsulation Efficiency (%)	90–98% mRNA encapsulation via microfluidic formulation	60–80% loading; limited by crystalline lipid matrix	80–95% improved loading due to amorphous liquid lipid core	50–75% RNA encapsulation; difficult direct condensation	5–20% inherently low mRNA loading capacity	[129,131,132,139]
RNA Protection Against RNase	High lipid bilayer protects RNA from enzymatic degradation	Moderate solid lipid matrix slows enzymatic access	High liquid lipid core improves cargo retention	Moderate polymer protects RNA but is vulnerable to hydrolysis	High endogenous lipid bilayer protects RNA cargo	[129,131,132,139]
Endosomal Escape Efficiency	Low (1–2%) primary bottleneck; requires ionizable lipid protonation	Low rigid lipid matrix limits membrane disruption	Moderate liquid lipid phase improves membrane fusion	Low polymer particles require escape modifiers	High (>20%) 10× higher than LNPs via natural fusion	[25,124,137,139]
Immunogenicity Profile	Moderate-High activates TLR4; risk of systemic inflammation	Low physiologically compatible lipids	Low similar lipid composition to SLNs	Low-moderate acidic degradation can trigger mild response	Lowest natural origin ensures minimal immune activation	[61,131,137,139]
In Vivo Transfection Efficiency	Highest strong systemic protein expression (ApoE/LDLR)	Moderate local delivery more effective	Moderate-High improved delivery vs. SLNs	Low limited by cytosolic release bottleneck	Moderate targeting depends on donor cell tropism	[87,129,131,139]

## Data Availability

No new data were created or analyzed in this study. Data sharing is not applicable to this article.
